# Carboxypeptidase E-ΔN, a Neuroprotein Transiently Expressed during Development Protects Embryonic Neurons against Glutamate Neurotoxicity

**DOI:** 10.1371/journal.pone.0112996

**Published:** 2014-11-26

**Authors:** Xiao-Yan Qin, Yong Cheng, Saravana R. K. Murthy, Prabhuanand Selvaraj, Y. Peng Loh

**Affiliations:** 1 Section on Cellular Neurobiology, Program on Developmental Neuroscience, Eunice Kennedy Shriver National Institute of Child Health and Human Development, National Institutes of Health, Bethesda, Maryland 20892, United States of America; 2 College of Life and Environmental Sciences, Minzu University of China, Beijing 100081, China; University of Louisville, United States of America

## Abstract

Neuroprotective proteins expressed in the fetus play a critical role during early embryonic neurodevelopment, especially during maternal exposure to alcohol and drugs that cause stress, glutamate neuroexcitotoxicity, and damage to the fetal brain, if prolonged. We have identified a novel protein, carboxypeptidase E-ΔN (CPE-ΔN), which is a splice variant of CPE that has neuroprotective effects on embryonic neurons. CPE-ΔN is transiently expressed in mouse embryos from embryonic day 5.5 to postnatal day 1. It is expressed in embryonic neurons, but not in 3 week or older mouse brains, suggesting a function primarily *in utero*. CPE-ΔN expression was up-regulated in embryonic hippocampal neurons in response to dexamethasone treatment. CPE-ΔN transduced into rat embryonic cortical and hippocampal neurons protected them from glutamate- and H_2_O_2_-induced cell death. When transduced into embryonic cortical neurons, CPE-ΔN was found in the nucleus and enhanced the transcription of FGF2 mRNA. Embryonic cortical neurons challenged with glutamate resulted in attenuated FGF2 levels and cell death, but CPE-ΔN transduced neurons treated in the same manner showed increased FGF2 expression and normal viability. This neuroprotective effect of CPE-ΔN was mediated by secreted FGF2. Through receptor signaling, FGF2 activated the AKT and ERK signaling pathways, which in turn increased BCL-2 expression. This led to inhibition of caspase-3 activity and cell survival.

## Introduction

Glutamate release is required for normal fetal neurodevelopment, but excess release leads to neuronal damage and neuropathogenesis. Various conditions such as maternal acute alcohol exposure *in vivo* [1], maternal hemorrhage [2] or maternal chronic hypoxemia [3], results in increased glutamate release from the fetal cortex leading to neuronal cell death. Endogenous neuroprotective factors exist in the fetal brain to maintain neuronal survival and prevent onset of apoptosis in response to stresses such as glutamate neurotoxicity, oxidative stress and ischemia [4], [5]. However, prolonged insult would lead to neuronal degeneration [6]. Neuroprotective agents expressed in the fetal brain include trophic factors such as brain-derived neurotrophic factor (BDNF) [7], [8], activity-dependent neuroprotective protein (ADNP) [9] and carboxypeptidase E (CPE)/neurotrophic factor-α1 (NF-α1) [9]–[11]. Various synthetic neuropeptides, such as the nonapeptide, activity-dependent neurotrophic factor 9 (ADNF 9) [12], [13], and NAP, found within the ADNP sequence [14], [15], have also been shown to have neuroprotective effects. ADNP, ADNF 9 and NF-α1 have been found to protect embryonic neurons from glutamate neurotoxicity and oxidative stress induced cell death [9], [10], [12], [16]. BDNF was also shown to increase the neuronal response to glutamate in order to reduce excitotoxic apoptosis in primary neurons [16]. Recently, neuropeptides, ADNF 9 and NAP have been shown to prevent alcohol-induced apoptosis in fetal brain of mice [17]. Thus, neuroprotective agents play a critical role during development of the fetal nervous system *in utero* especially when the mother is exposed to alcohol and drugs causing neuroexcitotoxicity.

Recently, in preliminary studies, we found a novel splice variant of carboxypeptidase E (CPE), CPE-ΔN, which is expressed in mouse and rat embryonic neurons, with no detectable expression at the protein level in the adult mouse brain. In contrast, wild-type CPE, which functions as a prohormone/proneuropeptide processing enzyme, is expressed during embryonic development [18] and very highly expressed in the adult mouse and rat brain [19], [20]. This finding suggested that CPE-ΔN might have an important function primarily during embryonic neurodevelopment.

Interestingly, CPE-ΔN mRNA has been reported to be highly expressed in metastatic cancer cells such as in hepatocellular carcinoma (HCC), colorectal carcinoma and pheochromocytoma, but not in normal liver, colon and adrenal cells, respectively [21]. This is perhaps not surprising since many oncoproteins which are abundant in cancer cells are also highly expressed during embryonic development, with some being developmentally down-regulated in expression in normal adult tissue, such as NEDD9 and NEDD4 [22]–[28]. CPE-ΔN was found to be an inducer of metastasis and is a powerful biomarker for predicting future metastasis in patients with different types of cancers [21], [29]. CPE-ΔN differs from CPE in that it is N-terminally truncated and lacks a signal peptide. It is transported into the nucleus where it interacts with histone deacetylase 1/2 to activate the expression of the metastatic gene, *Nedd9* in HCC cells [21]. Thus, CPE-ΔN acts intracellularly to regulate gene transcription in the nucleus, unlike CPE, which, in addition to acting as a carboxypeptidase in the granules of the regulated secretory pathway, has recently been shown to function extracellularly as a neurotrophic factor in neuroprotection [10] and depression [30].

In the present study, we have investigated the developmental expression of CPE-ΔN in mouse embryos and its presence in postnatal and adult mouse brain. We determined if CPE-ΔN expression is up-regulated in embryonic neurons in response to dexamethasone, the synthetic form of the stress hormone, glucocorticoid. We then investigated if CPE-ΔN has a neuroprotective role in embryonic neurons, and if so, its mechanism of action. Our results show that CPE-ΔN is up-regulated in expression after treatment of embryonic neurons with dexamethasone, and neuroprotects these neurons against glutamate neuroexcitotoxicity and H_2_O_2_-induced oxidative stress. CPE-ΔN acts by increasing the transcription and secretion of fibroblast growth factor (FGF2). Secreted FGF2 binds to its extracellular receptor to increase the expression of BCL-2, a pro-survival protein, to mediate neuroprotection.

## Materials and Methods

### Animals

All animals were given food and water ad libitum in a humidity and temperature controlled room under a 12 h light∶dark cycle. All animal procedures were approved by the Animal Care and Use Committee, NICHD, NIH. All the timed pregnant mice (E5.5–E14.5, E17.5, and postnatal day 1 were generated by mating C57BL6 mice in our animal facility. Mice (3–12 weeks old) and pregnant rats were purchased from Taconic, Hudson, NY. Mice were euthanized by cervical dislocation and rats by CO_2_ anesthesia and the animals were immediately decapitated. All pups were removed from the mother placed on ice and then dissected.

### Preparation of mouse E13.5 cortical cells

A timed pregnant mouse at gestational day 13.5 was sacrificed by cervical dislocation and decapitated. Embryos were removed and the brain cortices dissected making sure no meninges remained with the cortices. The cortices were digested with 1 ml of 0.05% trypsin (Gibco) for 5 min at 37°C, followed by neutralizing the trypsin with an equal volume of soybean trypsin inhibitor (Sigma Aldrich). The digested cortices were triturated by a pipette to make a homogenous mixture, which was then passed through a cell strainer (40 µm) (BD-Falcon) to remove undissociated tissue. The cells were then centrifuged for 5 min at 1800 rpm and the supernatant discarded. The cell pellet was resuspended in DMEM containing 1× antibiotics (Penicillin-Streptomycin) and 10% FBS and counted.

### Mouse embryonic stem cells

Mouse embryonic stem cells were obtained from ATCC [R1 (ATCC SCRC-1011)] and grown in flasks pre-plated with mouse embryonic fibroblasts (Gibco) in ES cell basal media (ATCC SCRR-2011) that was supplemented with 0.1 mM 2-mercaptoethanol (Life Technologies), 1,000 U/mL mouse leukemia inhibitory factor (Millipore) and 10% to 15% ES-Cell Qualified FBS (ATCC SCRR-30-2020), or an ES cell qualified serum replacement. Cells were extracted for total RNA and analyzed.

### Rat primary neuronal cultures

E18 embryos were obtained from rats and their brains removed. Hippocampal or cortical neuronal cultures were prepared as described previously [10]. Briefly, the tissue was dissected and digested in 2 ml of 2 mg/ml papain for 30 min at 37°C, which was then inactivated by 10% FBS (3 ml). The tissue was triturated by a pipette to make a homogenous mixture, which was then passed through a cell strainer to remove undissociated tissue. The cells were then centrifuged for 5 min at 1500 rpm, and the supernatant discarded. The cell pellet was resuspended in DMEM containing 1× antibiotics (Penicillin-Streptomycin) and 5% FBS. The cells were then plated on poly-L-lysine (Sigma) coated plates or coverslips at a density of 1×10^6^ cells/ml. The medium was replaced by Neurobasal medium with 2% B27 (Invitrogen) after plating over-night.

### Treatment of rat primary neurons

Rat hippocampal or cortical neurons were treated with 1 µM dexamethasone (Sigma) for 24 h. The cells were then harvested, extracted and analyzed for expression of CPE-ΔN. FGF2 (R&D) or BDNF (Promega) was added to neurons for 15 min. The cells were then harvested, extracted and analyzed for activation of ERK and AKT. In other experiments, primary neurons were transduced with adenoviral vectors (Type 5 (dE1/E3)), carrying the cDNA of CPE ΔN (custom made by Vector Biolabs) or LacZ (Vector Biolabs) as a negative control, at 50 MOI for 72 h. The neurons were then treated with 100 µM H_2_O_2_ (Sigma), 40 µM glutamate (Sigma) or 0.4 µM staurosporine (Sigma) for 24 h. In specific experiments, 10 µM LY294002 (Cell Signaling), 5 µM U0126 (Cell Signaling), 1 µM PD166285 (Sigma) or SU5402 (Sigma) were added to the neurons during the 20–24 h treatment with glutamate, staurosporine or H_2_O_2_. Cell viability, cell cytotoxicity and TUNEL assays were then used to measure cell death after the treatments.

### Preparation of nuclear and cytoplasmic fractions from rat primary neurons

Preparation and extraction of cytoplasmic and nuclear fractions from primary cultured cortical neurons was achieved using the NE-PER Nuclear and Cytoplasmic Extraction Kit according to the manufacturer's instructions (Thermo Scientific).

### Measurement of secreted FGF2

Secreted FGF2 in primary cultured cortical neurons was measured using a rat FGF2 ELISA kit according to the manufacturer's instructions (Cloud-Clone Corp).

### Western blot

Protein lysates of rat cortical or hippocampal neurons in culture were prepared by homogenizing with T-protein extraction reagent (T-per, Pierce) supplemented with 1× Complete Inhibitor Cocktail (Roche). The lysates were collected, centrifuged at 15,000 rpm for 10 min at 4°C and the protein concentrations from supernatants determined. To assay for CPE-ΔN protein, neurons in culture or brain tissue were dissolved with Novex 1× LDS sample buffer supplemented with Novex reducing agent (Life Technologies). Twenty micrograms of protein from the lysates were denatured at 70°C for 10 min or 20 µl of the dissolved cells or tissue were denatured at 90°C for 5 min and run on 4–20% or 12% SDS-PAGE gels and then transferred onto nitrocellulose membrane (Millipore), according to standard protocols. After blocking with 5% nonfat milk at room temperature for 1 h, the membrane was incubated with the primary antibodies overnight diluted in PBS supplemented with 0.1% Tween 20. After washing, the membrane was incubated with fluorophore conjugated anti-mouse or anti-rabbit antibodies (Amersham) and visualized by the Odyssey infrared imaging system version 2.1 (LI-COR Inc.). Bands were quantified by Odyssey software. The protein expression level for each sample was normalized with β-actin. Antibodies used: Monoclonal rabbit anti-cleaved active caspase-3 antibody (1∶3000), monoclonal mouse anti-p-AKT antibody (1∶3000), polyclonal rabbit anti-t-AKT antibody (1∶3000), polyclonal rabbit anti-t-ERK antibody (1∶3000) and polyclonal rabbit anti-Bcl-2 antibody (1∶3000) were from Cell Signaling. Monoclonal mouse anti-p-ERK antibody (1∶1000) was from Santa Cruz. Polyclonal rabbit anti-acetylated histone 3 antibody was from Active Motif. Mouse monoclonal beta-tubulin antibody was from BD Pharmingen. Polyclonal rabbit anti-FGF2 (1∶3000) antibody was from Abcam and purified polyclonal rabbit anti-CPE antibody (#6135) [21], directed to an internal sequence of CPE, was generated in our laboratory. Mouse monoclonal CPE antibody was purchased from BD Biosciences.

### Semi-quantitative PCR of CPE and CPE-ΔN transcripts in E13.5 cortical cells and embryonic stem cells (ESC)

RNA was extracted from E13.5 cortical cells (2×10^6^ cells) and embryonic stem cells (ESC) (2×10^6^ cells) using Promega SV RNA isolation kit (Promega) according to manufacturer's instructions and cDNA was synthesized with 500 ng of total RNA from these cells using Transcriptor first strand cDNA synthesis kit (Roche Applied Science). Semi-quantitative PCR was performed to quantify CPE-ΔN transcripts using GoTaq Green Master Mix (Promega). 18S rRNA was used for normalization. Primer sequences specific for the mouse CPE-ΔN fwd: 5′-GACAAAAGAGGCCAGCAAGA-3′, and CPE-ΔN rev: 5′-CAGGTTCACCCGGCTCAT-3′; for 18S fwd: 5′-CTCTTAGCTGAGTGTCCCGC-3′, rev: 5′-CTGATCGTCTTCGAACCTCC-3′. One µl of cDNA was used for every reaction. PCR cycling was at 94°C for 15 s, annealing at 60°C for 30 s, extension at 72°C for 30 s, and a final extension at 72°C for 10 min. Fifteen microliters of each sample was removed every 5 cycles from 25 to 35 cycles in each reaction to amplify CPE-ΔN and 18S fragments. Amplified PCR products were separated on 1.6% agarose gels with Tris-borate EDTA buffer and stained with ethidium bromide. Gels were captured as digital images and the bands quantified by densitometry (Image J).

### Quantitative RT-PCR

#### Mouse embryos

Total RNA was isolated from mouse whole embryo or head lysates using Promega SV RNA isolation kit according to the manufacturer's instructions. First strand complementary DNA (cDNA) was prepared from 500 ng of total RNA using Transcriptor first strand cDNA synthesis kit (Roche Applied Science).

#### Mouse brain and rat embryonic primary neurons

Total RNA was extracted from postnatal day 1, 3, 4, 6 and 12 week old mouse hippocampus and cortex, and primary cultures of embryonic cortical or hippocampal neurons using Trizol (Invitrogen) and chloroform, and purified using the RNeasy mini kit (Qiagen) and quantified. First strand cDNAs were synthesized with 500 ng of RNA using Transcriptor first strand cDNA synthesis kit (Roche Applied Science).

#### PCR amplification

PCR amplification was carried out with 100 nM (18S rRNA) or 300 nM (Bcl-2 or Fgf2) of forward and reverse primers, in a 12.5 µl volume, in an ABI 7500 Sequence Detector (Applied Biosystems). The cycling conditions were: 10 min denaturation at 95°C and 40 cycles of DNA synthesis at 95°C for 15 s and 60°C for 1 min. Primer sequences for rat CPE-ΔN fwd: 5′-CAAAAGAGACCAGCAGGAGGAC-3′, rev: 5′-TCAGGTTCACCGGGCTCATG-3′; mouse CPE-ΔN fwd: 5′-GACAAAAGAGGCCAGCAAGA-3′, rev: 5′-CAGGTTCACCCGGCTCAT-3′; rat Bcl2 fwd: 5′-AAGCTGTCACAGAGGGGCTA-3′, rev:5′CAGGCTGGAAGGAGAAGATG-3′; rat FGF2 fwd: 5′- CACTTACCGGTCACGGAAAT-3′, rev: 5′- CCGTTTTGGATCCGAGTTTA-3′; for 18S-fwd: 5′-CTCTTAGCTGAGTGTCCCGC-3′, rev: 5′-CTGATCGTCTTCGAACCTCC-3′. Fluorescence signals were analyzed using SDS 1.9.1 software (Applied Biosystems). 18S rRNA was used as the endogenous control for normalization. All qPCRs were performed in triplicates and were averaged to obtain the data point for each specimen. The relative amount of CPE mRNA was normalized to an internal control, 18S rRNA, given by Livak and Schmittgen [31], 2^−ΔΔCT^, where ΔΔCT = [CT(CPE-ΔN)−CT(18S)]test−[CT(CPE-ΔN)−CT(18S)]. The threshold value (CT) was defined as the fractional cycle number at which the amount of amplified target reached a fixed threshold.

### WST-1 assay for cell viability

The viability of the cells was determined by the WST-1 Cell Proliferation Reagent (Clonetech) assay in a 96 well plate. The WST assay is a water soluble version of the standard MTT assay for cell viability. After the different treatments, 10 µl of premixed WST-1 was added to each well and the plate was maintained in a 37°C incubator for 1–1.5 h. The absorbance of the samples was then measured at 440 nm using a multi-well plate reader.

### Lactic Dehydrogenase (LDH) release assay for cell cytotoxicity

The cytotoxicity of cells after various treatments was evaluated by the extent of the release of LDH. This was achieved with a CytoTox 96 Non-Radioactive Cytotoxicity Assay kit according to the manufacturer's instructions (Promega).

### TUNEL assay

TUNEL assay was performed as described previously. Briefly, primary cultured cortical neurons grown on slides were fixed and permeabilized after the various treatments. An *in situ* cell death detection kit, TUNEL (Roche), was used to stain the cells. After staining, coverslips were mounted over the slides which were then imaged under the fluorescence microscope. The cell nuclei were stained with DAPI. The percentage of cell death was determined by the ratio of the number of TUNEL-positive cells over the total DAPI stained cells. At least 500 cells were counted in each well. The average of 3 wells was calculated as the percentage of neuronal cell death after the various treatments.

### Immunocytochemistry of primary neurons

Primary rat cortical neurons were treated under different experimental conditions (cultured in chambers) and then gently washed with 1× PBS before being fixed with 4% paraformaldehyde in PBS. Cells were then washed with PBS and blocked using PBS containing 10% goat serum and 0.3% triton. With the fixation solution removed, the cells were incubated with primary antibody (rabbit polyclonal CPE antibody #6135) diluted in 3% serum-PBS at 4°C overnight. The cells were then rinsed and incubated with secondary antibody (Alexa Fluor 594 goat anti-rabbit secondary antibody from Invitrogen) for 1 h at RT. After washing with PBS, the chambers were mounted with mounting medium containing DAPI. Stained neurons were then visualized and photographed under a fluorescence microscope.

### Statistical analysis

Data were analyzed by Student's *t*-test and one-way analysis of variance (ANOVA) followed by Tukey post-hoc multiple comparisons tests where noted. Significance was set at p<0.05.

## Results

### CPE-ΔN is expressed in embryos and embryonic neurons but not adult brain

**Figure 1:**
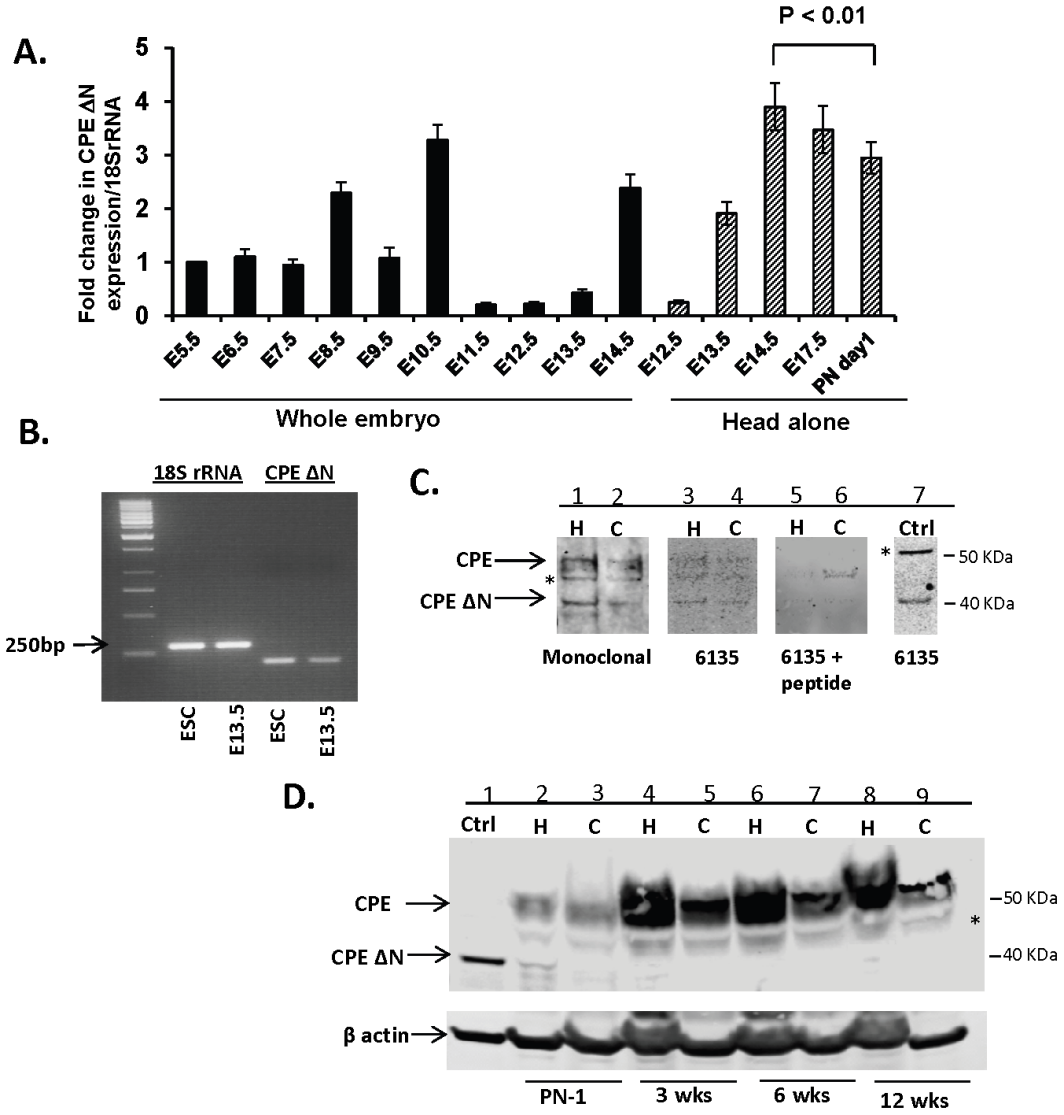
Expression of CPE-ΔN in embryos, embryonic neurons and adult brain. A. Bar graph represents the fold change in *CPE-ΔN* mRNA levels in whole mouse embryos and head alone at gestational ages from E5.5–E17.5 and postnatal day 1 (PN1) compared to E5.5. Each bar represents results from 3 embryos or head, each one obtained from 3 individual mice. The RT-PCR was done in triplicates. Note the significant decrease in the *CPE-ΔN* mRNA levels from head of E14.5 embryos to PN1 mice (*t* test, n = 3, p<0.01). B. Semi-quantitative PCR of *CPE-ΔN* transcripts. Representative gel (n = 3) showing 18S rRNA and 200 bp *CPE-ΔN* transcripts from mouse E13.5 cortical cells and embryonic stem cells (ESC). C. Representative Western blot showing WT and CPE-ΔN protein in rat E18 hippocampal (H) and cortical (C) neurons visualized by 2 different antibodies: mouse monoclonal antibody, (lanes 1, 2), rabbit polyclonal antibody #6135 (lanes 3, 4) and absorption control using the antigenic peptide used to generate antibody #6135 (lanes 5, 6). A positive control (Ctrl) using HCC cells transduced with *cpe-ΔN* construct (lane 7). * denotes non-specific band. N = 3. D. Representative Western blot (N = 3) showing the expression of CPE-ΔN and CPE from hippocampus (H) (lanes: 2, 4, 6, 8) and cortex (C) (lanes 3, 5, 7, 9) of mice at PN1, 3, 6 and 12 weeks of age and positive control (Ctrl. using HCC cells transduced with cpe-*ΔN* construct) (lane 1), probed with mouse monoclonal antibody. * denotes non-specific band.

*CPE-ΔN* mRNA was analyzed in mouse embryos from embryonic day 5.5 (E5.5) to 17.5 (E17.5) and postnatal day 1 (PN1). Fig. 1A shows the temporal profile of expression of *CPE-ΔN* mRNA relative to expression at E5.5 in whole embryos and in the head during later gestational ages. In whole embryos, *CPE-ΔN* mRNA was detected at E5.5 and the amount doubled at E8.5. It then decreased at E9.5, followed by a 3-fold increase at E10.5. Between E11.5 and 13.5, expression fell below the E5.5 level, but increased sharply at E14.5. The levels in the head showed an increase between E12.5 and E14.5 and then declined through PN1. Comparing relative *CPE-ΔN* mRNA levels in whole embryos versus the head at E13.5 and E14.5, it is evident that most of the expression was in the head, which primarily contains brain tissue. Semi-quantitative PCR revealed the expression of *CPE-ΔN* transcripts (Fig. 1B) in E13.5 mouse cortical cells and embryonic stem cells (ESC).

Western blot analysis showed low levels of expression of CPE-ΔN protein in rat E18 embryonic hippocampal and cortical neurons (Fig. 1C). CPE-ΔN protein was also found in the hippocampus, but not detectable in the cortex of mice at PN1. No CPE-ΔN was detected in the hippocampus or cortex of 3, 6 and 12 week old mice (Fig. 1D). These results indicate that there is a down-regulation of CPE-ΔN protein expression in mouse brain after birth. This is in contrast to CPE, which is highly expressed in the hippocampus and cortex of adult mice (Fig. 1D).

**Figure 2:**
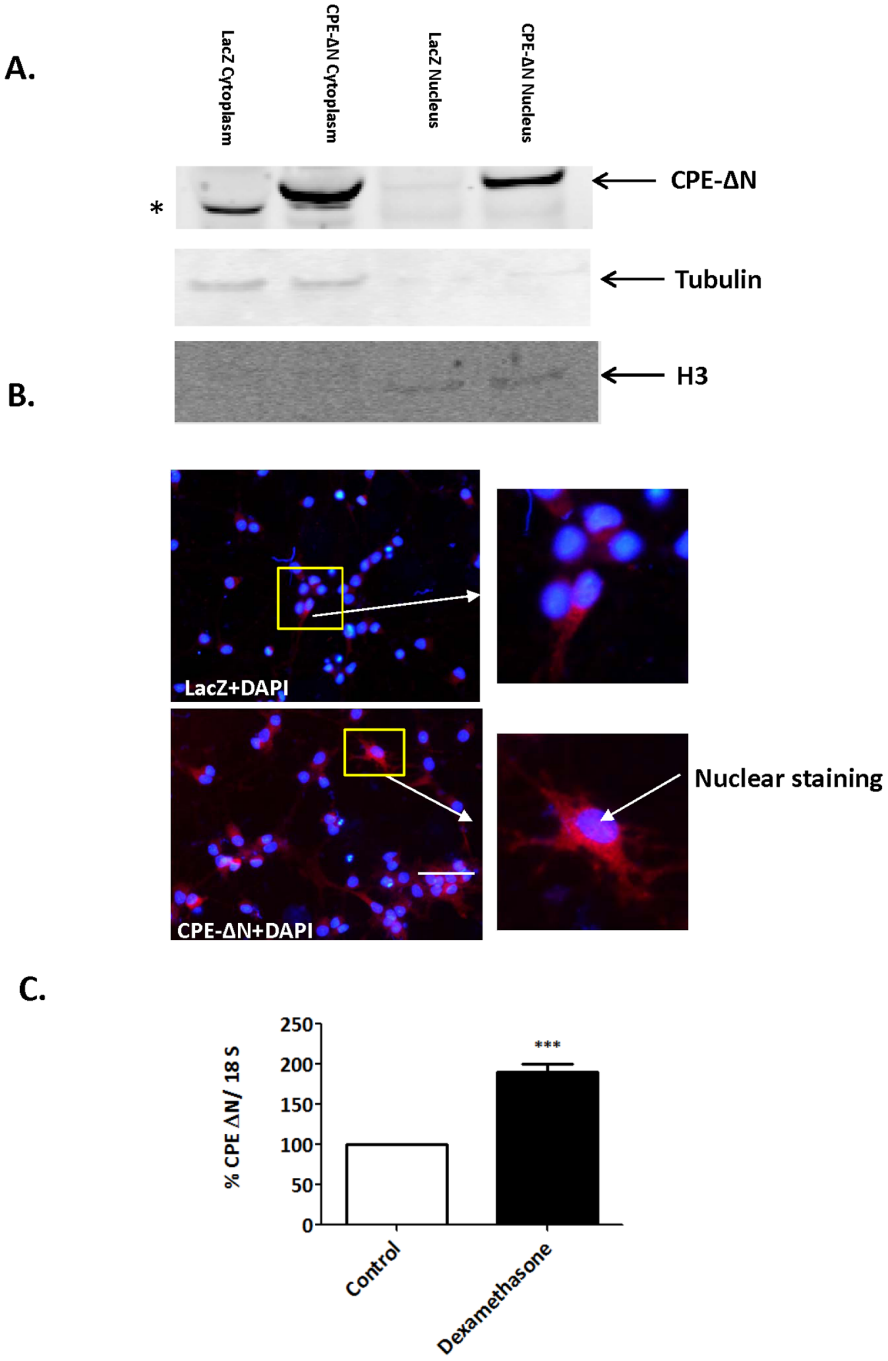
Subcellular localization and up-regulation of CPE-ΔN mRNA expression by dexamethasone. A. Cellular localization of CPE-ΔN in rat E18 cortical neurons transduced with adenovirus carrying CPE-ΔN construct. Western blot shows the presence of CPE-ΔN in the cytoplasm and the nucleus of CPE-ΔN-transduced cells, but not visible in the LacZ-transduced control cells. Beta-tubulin served as a marker for cytoplasm and Histone-3 served as a marker for nucleus. * indicate a non specific band in the cytoplasm. B. Immunocytochemistry confirm CPE-ΔN (red) immunostaining in the nucleus marked by DAPI staining (blue) and is more visible in the CPE-ΔN-transduced vs. control cells. Insets show a higher magnification of cells indicated by the arrow. Bar = 50 microns. C. Bar graphs show an increase in *CPE-ΔN* mRNA expression in rat E18 hippocampal neurons treated with dexamethasone compared to control untreated neurons. Values are the mean ±SEM (n = 4). *t* test *** p<0.001.

To determine the cellular localization of CPE-ΔN, nuclear and cytoplasmic fractions from E18 rat cortical neurons transduced with adenovirus carrying the *CPE-ΔN* construct were prepared. The Western blot in Fig. 2A shows that CPE-ΔN is localized in both the cytoplasm and the nucleus. Immunocytochemical studies confirmed the presence of CPE-ΔN in the nucleus of hippocampal neurons (Fig. 2B, bottom panels).

To investigate if *CPE-ΔN* mRNA expression could be up-regulated during stress, E18 hippocampal neurons in culture were treated with dexamethasone. Fig. 2C shows that *CPE-ΔN* mRNA expression was increased with dexamethasone treatment.

### CPE-ΔN protects cortical neurons against glutamate-induced neurotoxicity

**Figure 3:**
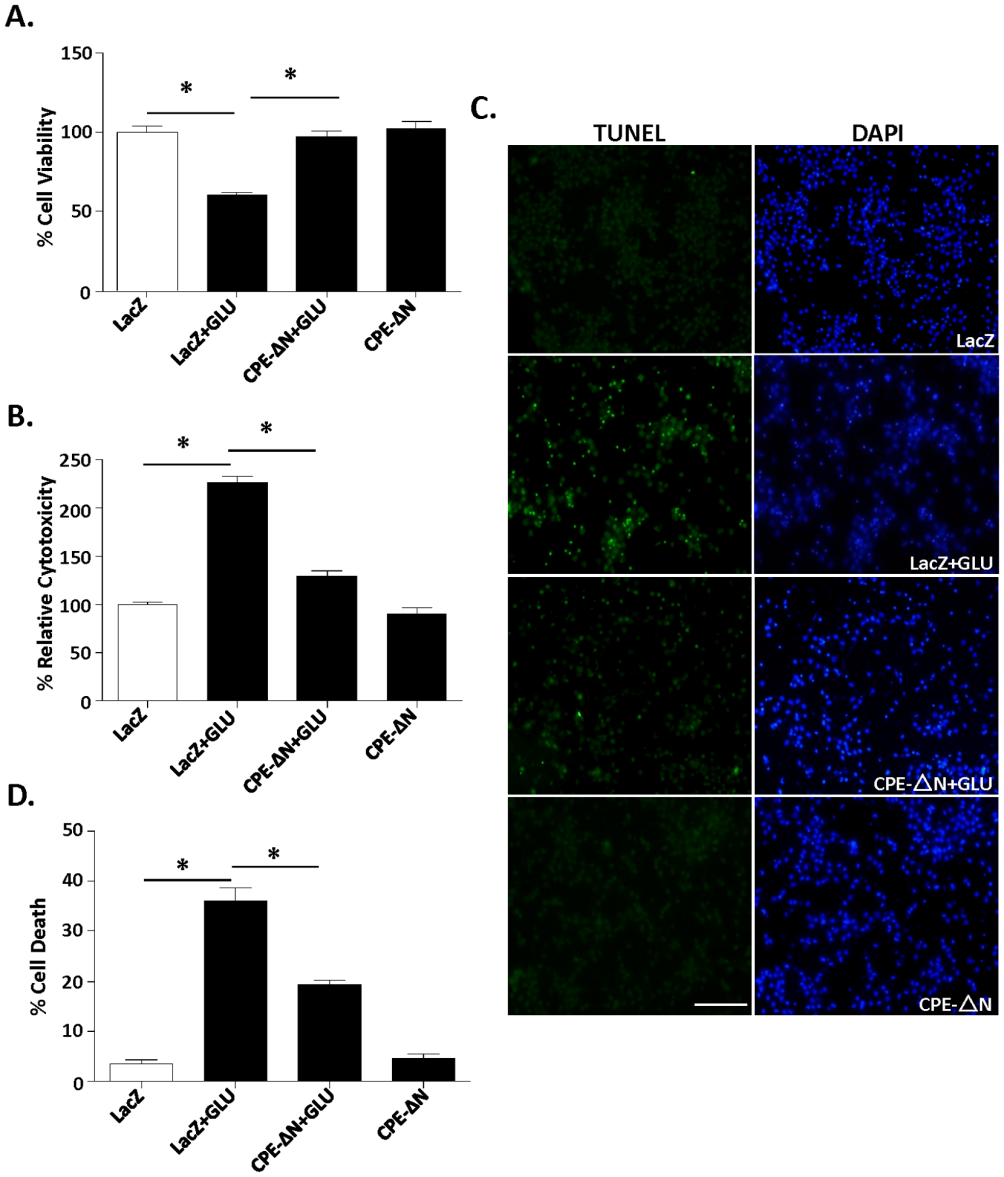
CPE-ΔN protects primary cortical neurons against glutamate-induced neurotoxicity. In A–D, rat primary cortical neurons were transduced with CPE-ΔN or LacZ viral vectors and subsequently treated with or without with glutamate for 24 h. A. Bar graphs show WST activity, indicative of cell viability. Note that the reduced cell viability after glutamate treatment was significantly increased in neurons transduced with the CPE-ΔN construct. At least three independent experiments were done. Data shown represent one experiment. B. Bar graphs show LDH release, indicative of cytotoxicity of cortical neurons. Note that the glutamate-induced cytotoxicity was significantly attenuated by the transduction of CPE-ΔN construct. At least three independent experiments were done. Data shown represent one experiment. C, D. Photomicrographs and bar graphs showing cortical neurons stained with TUNEL (green) and DAPI (blue). Note that the number of dead cells (green) increased significantly after glutamate treatment and that transduction of CPE-ΔN construct protected the neurons. The bar graphs represent the quantification of dead cells as a % of the total number of cells determined by the DAPI staining. At least 500 cells were counted in each of 3 different dishes generated from embryos from two independent experiments. Data shown represent one experiment. Bar = 100 microns, n = 6/group (A); 5/group (B) and 3/group (C). Values are mean ± SEM, one-way ANOVA followed by Tukey test, *p<0.05.

To determine if CPE-ΔN has neuroprotective activity, cortical neurons from E18 rats were transduced with adenovirus carrying the LacZ (control) or *CPE-ΔN* constructs to overexpress these proteins, and then incubated with and without glutamate for 24 h. Our immunocytochemical results (Fig. 2B) showed that 15.1% (15.1±1.536%, n = 5) of neurons were transduced.with *CPE-ΔN*. Neurons treated with glutamate exhibited poor cell viability (Fig. 3A) (ANOVA between LacZ control and CPE-ΔN transduced neurons with/out glutamate treated groups; F_(3,20)_ = 30.57, p<0.001), and enhanced cytotoxicity (Fig. 3B) (F_(3,16)_ = 128.8, p<0.001), compared to the control group. However, overexpression of CPE-ΔN prior to glutamate treatment protected these neurons from cell death (Fig. 3A, B). To further confirm the neuroprotective effect of CPE-ΔN, the TUNEL assay was used. Fig. 3C, D show that cell death was greatly increased in control neurons after glutamate treatment compared to untreated cells (F_(3,8)_ = 103.7, p<0.001); however cell death was significantly reduced in the neurons overexpressing CPE-ΔN.

Similar neuroprotective effects were observed in rat embryonic hippocampal neurons overexpressing CPE-ΔN with glutamate treatment (Fig. S1), and cortical neurons treated with H_2_O_2_ to induce oxidative stress (Fig. S2) or staurosporine, a protein kinase inhibitor (Fig. S3).

### Neuroprotection by CPE-ΔN is mediated by FGF2

**Figure 4:**
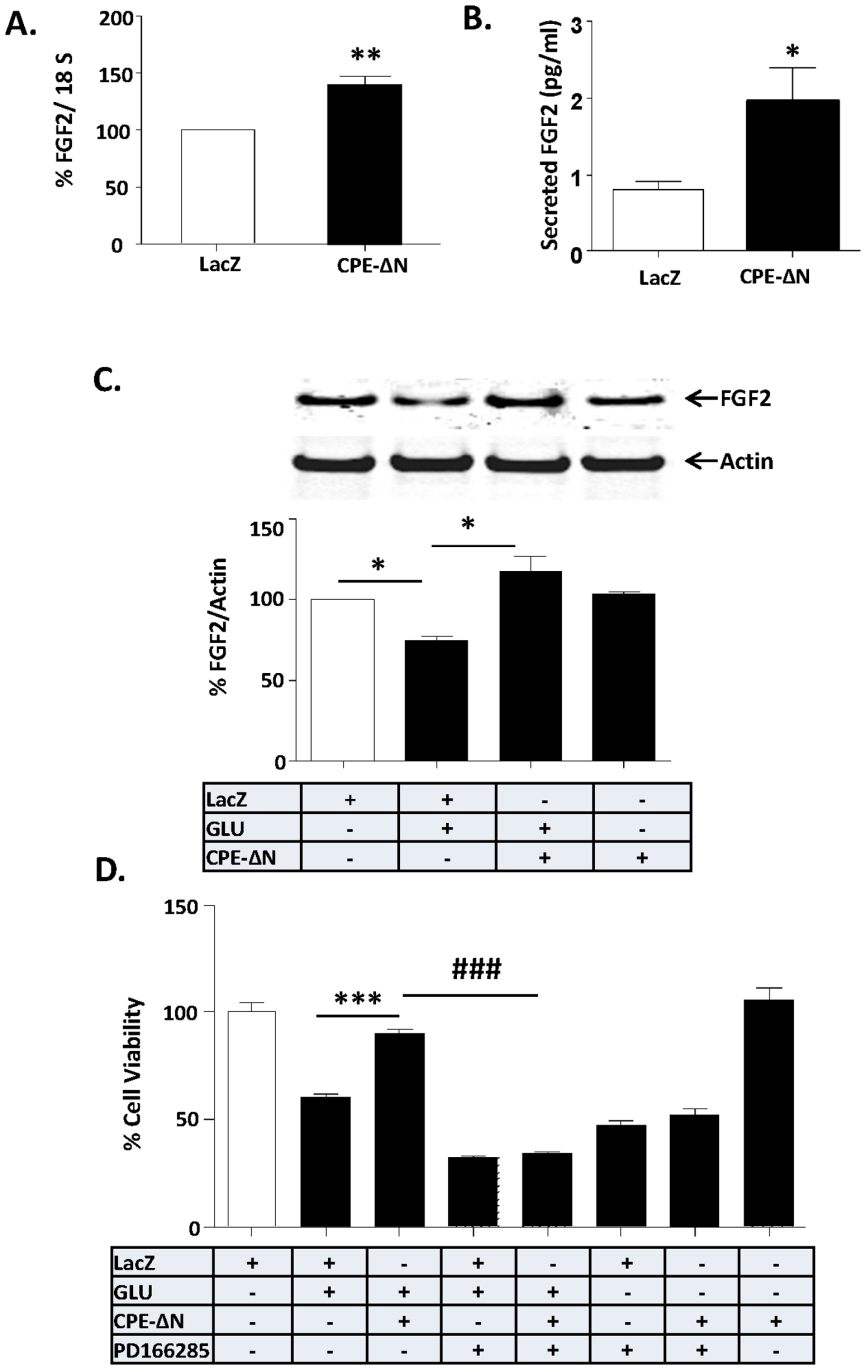
Neuroprotection by CPE-ΔN is mediated by FGF2. A. Bar graph shows the quantification by qRT-PCR of *FGF2* mRNA in primary cultured rat cortical neurons after transduction with CPE-ΔN or LacZ (control) viral vectors. Data are normalized against 18S RNA and presented as a % compared to control (LacZ) cells. Values are the mean ± SEM (n = 5), *t* test, ** p<0.01. At least three independent experiments were done. Data shown represent all the experiments combined. B. Bar graph shows the levels of secreted FGF2 by ELISA in primary cultured rat cortical neurons after transduction with CPE-ΔN or LacZ (control) viral vectors. Values are the mean ± SEM (n = 4), *t* test, * p<0.05. Two independent experiments were done. Data shown represent one experiment. C. Top panel: Western blot analysis of FGF2 protein in primary cortical neurons, transduced with CPE-ΔN or LacZ viral vectors and subsequently challenged with or without glutamate for 24 h. Actin was also analyzed and served as an internal control for protein load; Bottom panel: Bar graphs show the quantification of FGF2 protein normalized to actin and expressed as a % compared to vehicle treated control cells. Note that CPE-ΔN significantly inhibited the glutamate-induced decrease in FGF2 protein in primary cultured cortical neurons. At least three independent experiments were done. Data shown represent all the experiments combined. D. Bar graphs show WST activity, indicative of cell viability of rat cortical neurons with and without transduction of CPE-ΔN construct and treated with and without glutamate in the continued presence or absence of FGF receptor inhibitor, PD166285. Note the neuroprotective effect of CPE-ΔN was blocked by PD166285 in primary cultured cortical neurons, indicating that FGF2 mediates the neuroprotective effect of CPE-ΔN. Two independent experiments were done. Data shown represent one experiment. n = 6/group (C); 5/group (D). Values are mean ± SEM, one-way ANOVA followed by Tukey test, *p<0.05.

To elucidate the mechanism by which CPE-ΔN protects neurons, we searched for the activation of potential downstream target genes that are known to be involved in cell survival, since CPE-ΔN is present and functions in the nucleus to up-regulate gene transcription in cancer cells [21]. We tested if CPE-ΔN can up-regulate the expression of FGF2, a growth factor that promotes neuronal survival [32]. Figure 4A shows that rat primary cortical neurons transduced with CPE-ΔN showed increased expression of *FGF2* mRNA compared to control (LacZ transduced) cells. Moreover analysis of FGF2 in the cell culture medium showed increased secretion of FGF2 in cortical neurons transduced with CPE-ΔN (Fig. 4B). *CPE-ΔN*-transduced primary cortical neurons were then challenged with glutamate-induced neurotoxicity and their FGF2 protein levels were assayed by Western blot (Fig. 4C). Glutamate treatment resulted in a significant decrease in FGF2 levels compared to untreated control neurons. However, in CPE-ΔN-transduced neurons treated with glutamate, FGF2 levels were not decreased (Fig. 4C) (F_(3,20)_ = 137.1, p<0.001), but were similar to levels in control cells. In assessing cell viability, the decrease in FGF2 levels induced by glutamate treatment resulted in poor cell viability compared to control cells (Fig. 4D). However, in CPE-ΔN-transduced neurons treated with glutamate, there was no decrease in viability (Fig. 4D). To attribute this neuroprotective effect to the action of FGF2, the CPE-ΔN-transduced neurons were treated with glutamate in the presence of an FGF2 receptor (FGFR1) inhibitor, PD166285. The neuroprotective effect of CPE-ΔN was abolished by PD166285 indicating that FGF2 secreted into the medium mediates the neuroprotection (ANOVA between LacZ control and CPE-ΔN transduced neurons with/out glutamate or PD166285 treated groups; F_(7,32)_ = 102.3, p<0.001).

Similar results were obtained in CPE-ΔN-transduced neurons challenged with H_2_O_2_ to induce oxidative stress (Fig. S4) or staurosporine, a protein kinase inhibitor (Fig. S5). CPE-ΔN protected rat primary cortical neurons against cell death induced by these two stressors. Moreover, PD166285, an FGF2 receptor inhibitor, and SU5402, a highly specific FGF receptor inhibitor, both inhibited the neuroprotective effect of CPE-ΔN in these neurons. We verified the specificity of the PD166285 inhibitor by incubating primary cultured cortical neurons with 50 ng/ml FGF2 or BDNF in the absence or presence of PD166285 for 15 min. Our results showed that FGF2 -induced ERK and AKT activation were blocked by PD166285 (Fig. S6). However, PD166285 had no effect on BDNF induced ERK and AKT activation, suggesting the specificity of the inhibitor for FGF2.

### Neuroprotection by CPE-ΔN involves AKT and ERK signaling pathway activation

**Figure 5:**
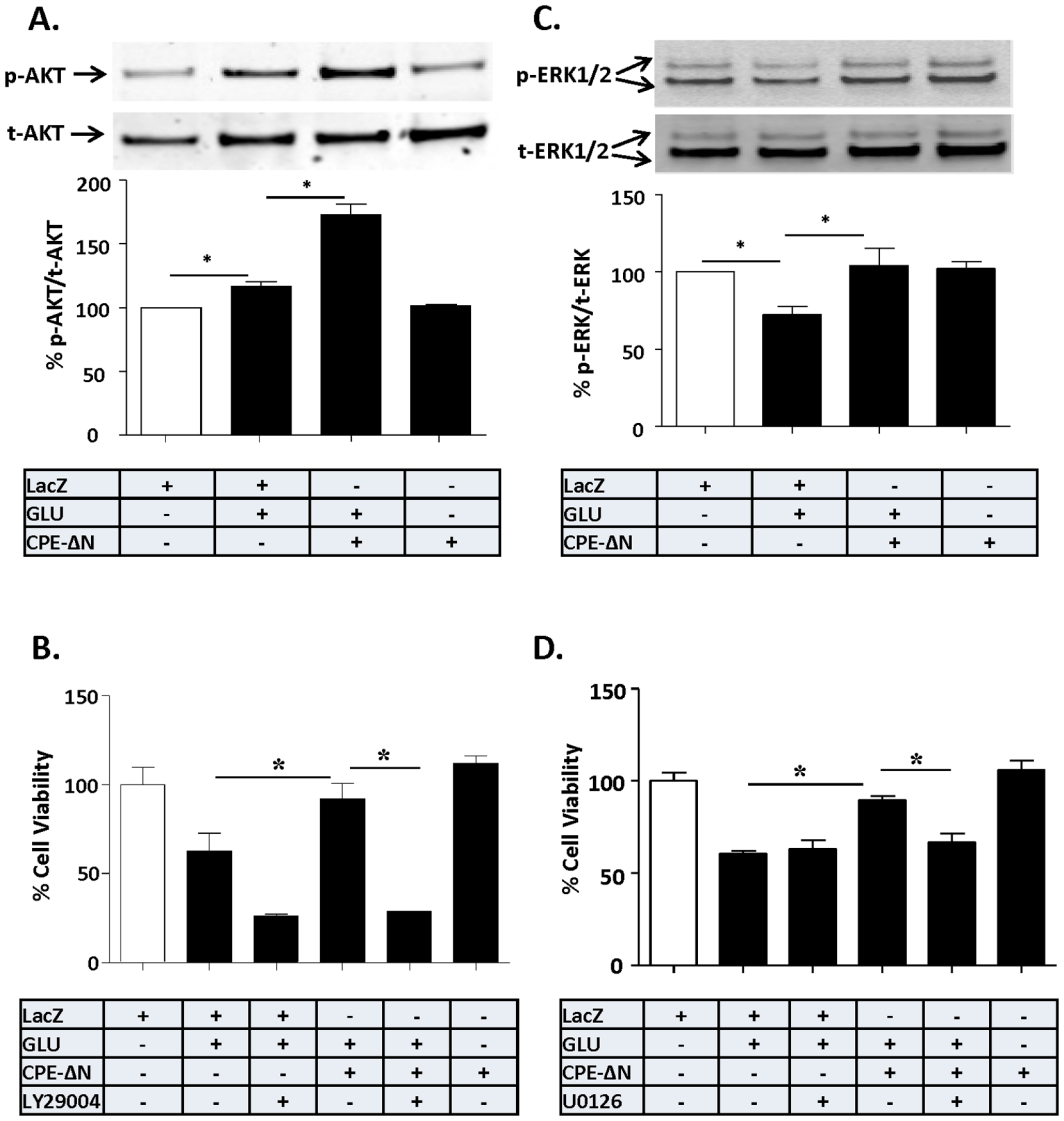
Neuroprotection by CPE-ΔN involves AKT and ERK signaling pathways. In A–D, rat primary cortical neurons were transduced with CPE-ΔN or LacZ vectors and subsequently challenged with or without glutamate for 24 h. A. Top panel: Western blot analysis of p-AKT protein in cortical neurons. Actin was also analyzed and served as an internal control for protein load. Bottom panel: Bar graphs showing the quantification of p-AKT normalized to t-AKT and expressed as a % compared to vehicle treated control cells. Note that CPE-ΔN significantly increased the level of p-AKT after the glutamate treatment in primary cultured cortical neurons. At least three independent experiments were done. Data shown represent all the experiments combined. B. Bar graphs showing WST activity, indicative of cell viability, of cortical neurons treated with and without glutamate in the continued presence or absence AKT inhibitor, LY294002. Note the neuroprotective effect of CPE-ΔN was completely blocked by LY294002, suggesting the AKT signaling pathway is involved in the neuroprotective effect of CPE-ΔN in primary cortical neurons. Two independent experiments were done. Data shown represent one experiment. C. Top panel: Western blot analysis of p-ERK in primary cortical neurons. Actin was also analyzed and served as an internal control for protein load; bottom panel: Bar graphs showing the quantification of p-ERK normalized to t-ERK and expressed as a % compared to vehicle treated control cells. Note that CPE-ΔN significantly inhibited the glutamate-induced decrease in p-ERK in primary cortical neurons. At least three independent experiments were done. Data shown represent all the experiments combined. D. Bar graphs showing WST activity, indicative of cell viability of cortical neurons treated with and without glutamate in the continued presence or absence of the ERK inhibitor, U0126. Note the neuroprotective effect of CPE-ΔN was partially blocked by U0126 suggesting the involvement of ERK signaling pathway in the cortical neurons. Two independent experiments were done. Data shown represent one experiment. n = 4/group (A); 5/group (B); 4/group (C); 5/group (D). Values are mean ± SEM, one-way ANOVA followed by Tukey test, *p<0.05.

Since CPE-ΔN-mediated neuroprotection is through FGF2 signaling which is known to involve AKT and ERK [32], we examined the activation of these two pathways in CPE-ΔN-transduced cortical neurons treated with glutamate. Fig. 5 shows that 24 h after glutamate treatment, there was a significant increase in phosphorylated AKT (Fig. 5A) (ANOVA between LacZ control and CPE-ΔN transduced neurons with/out glutamate treated groups; F_(3,12)_ = 83.96, p<0.001), and ERK 1/2 (Fig. 5C) (F_(3,20)_ = 4.958, p<0.01) in neurons overexpressing CPE-ΔN. To determine if AKT and/or ERK phosphorylation is required for the neuroprotective effect of CPE-ΔN, the PI3-K inhibitor, LY294002 and the MEK inhibitor, U0126 were applied to cortical neurons and cell viability were assessed after glutamate challenge. Fig. 5B (ANOVA between LacZ control and CPE-ΔN transduced neurons with/out glutamate or LY294002 treated groups; F_(5,24)_ = 28.13, p<0.001) and figure 5D (ANOVA between LacZ control and CPE-ΔN transduced neurons with/out glutamate or U0126 treated groups; F_(5,24)_ = 23.62, p<0.001) show that both inhibitors significantly blocked the neuroprotective effect of CPE-ΔN in neurons treated with glutamate.

Similar results were obtained in CPE-ΔN-transduced neurons challenged with H_2_O_2_ to induce oxidative stress (Fig. S7).

### Neuroprotective effect of CPE-ΔN is accompanied by an increase in BCL-2 expression and inhibition of caspase-3

**Figure 6:**
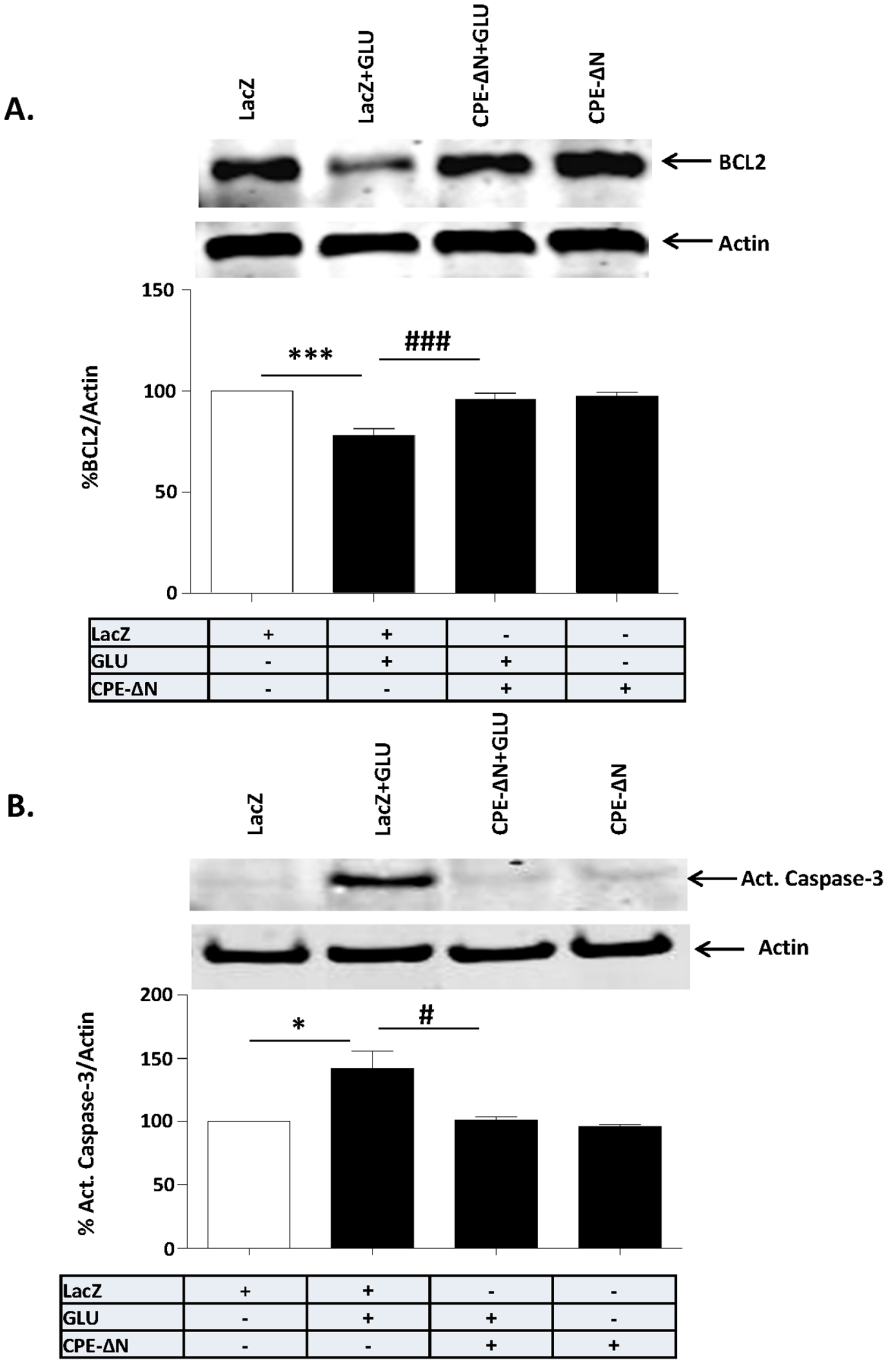
Neuroprotection by CPE-ΔN involves BCL-2 and Caspase-3. In A, B, rat primary cortical neurons were transduced with CPE-ΔN or LacZ viral vectors and subsequently treated with or without with glutamate for 24 h. A. Top panel: Western blot of BCL-2 protein in primary cortical neurons challenged or not with glutamate. Actin was also analyzed and served as an internal control for protein load; bottom panel: Bar graphs showing the quantification of BCL-2 normalized to actin and expressed as a % compared to vehicle treated control cells. Note that CPE-ΔN significantly inhibited the glutamate-induced decrease in BCL-2 protein in the cortical neurons. At least three independent experiments were done. Data shown represent all the experiments combined. B. Top panel: Western blot of caspase 3 protein in primary cortical neurons challenged or not with glutamate. Actin was also analyzed and served as an internal control for protein load; Bottom panel: Bar graphs showing the quantification of active caspase-3 normalized to actin and expressed as a % compared to vehicle treated control cells. Note that CPE-ΔN significantly inhibited the glutamate-induced activation of caspase-3 in the primary cortical neurons. At least three independent experiments were done. Data shown represent all the experiments combined. n = 6/group (A); 3/group (B). Values are mean ± SEM, one-way ANOVA followed by Tukey test, *p<0.05.

To further investigate the mechanism of action, we analyzed the expression of the anti-apoptotic protein, BCL-2, which is a downstream target of the AKT signaling pathway, in cortical neurons with and without glutamate treatment. Western blot and quantification of BCL-2 (Fig. 6A) show that cortical neurons treated with glutamate have decreased levels of BCL-2, but in CPE-ΔN- transduced neurons, the level was similar to cells that were not treated with glutamate (Fig. 6A) (ANOVA between LacZ control and CPE-ΔN transduced neurons with/out glutamate treated groups; F_(3,20)_ = 15.85, p<0.001). Additionally, we show that the activation of caspase-3 which was induced by glutamate treatment did not occur in CPE-ΔN-transduced cortical neurons (Fig. 6B) (F_(3,8)_ = 9.206, p<0.01).

## Discussion

In this study, we have identified the splice variant of CPE, CPE-ΔN, as a new neuroprotective protein that likely plays an important role specifically during embryonic development. We found that *CPE-ΔN* mRNA is expressed in mouse embryos as early as E5.5 and in embryonic stem cells. The level of *CPE-ΔN* mRNA expression varied throughout gestation, showing episodic expression levels at E8.5, E10.5 and E13.5–17.5. Comparison of the relative expression of *CPE-ΔN* mRNA in whole embryos and the head at E13.5 indicated that most of the transcripts were present in the head, and presumably in the brain. Within the early phase of mouse embryonic nervous system development, between E8.5–E10.5, there is massive expansion of neuroprogenitor cells [33] and CPE-ΔN might be involved in this proliferation phase, as was demonstrated for cancer cell proliferation [21]. At E11.5, neuroprogenitor cells begin to switch to neurogenesis, peaking at E13.5–E14.5 [34], [35]. Whether the surge in *CPE-ΔN* expression from E13.5–E14.5 is involved in this differentiation event remains to be determined. Western blot analysis indicated the translation of *CPE-ΔN* mRNA to protein in embryos, as seen in rat E18 hippocampal and cortical neurons in culture (Fig. 1C). Expression of CPE-ΔN protein was also observed in the mouse hippocampus at PN1, but was down-regulated by 3 weeks, with no detectable CPE-ΔN protein in the hippocampus or cortex of 3–12 week old mice. This is in contrast to CPE, which is highly expressed in the adult mouse brain (Fig. 1D). These findings support a specific role of CPE-ΔN in embryonic nervous system function, but not in adult brain. Suffice to say, CPE-ΔN is expressed in adults primarily during pathogenesis, as in various metastatic tumors [21], including gliomas [36]. However, some *CPE-ΔN* mRNA has been detected in stem cells isolated from the subventricular zone of adult mouse brain (our unpublished data). The expression pattern of *CPE-ΔN* during embryonic development may be similar to ADNP which has been reported to play a critical role for brain formation [37]. Thus *CPE-ΔN* and ADNP may have a coordinated role during embryonic development.

**Figure 7:**
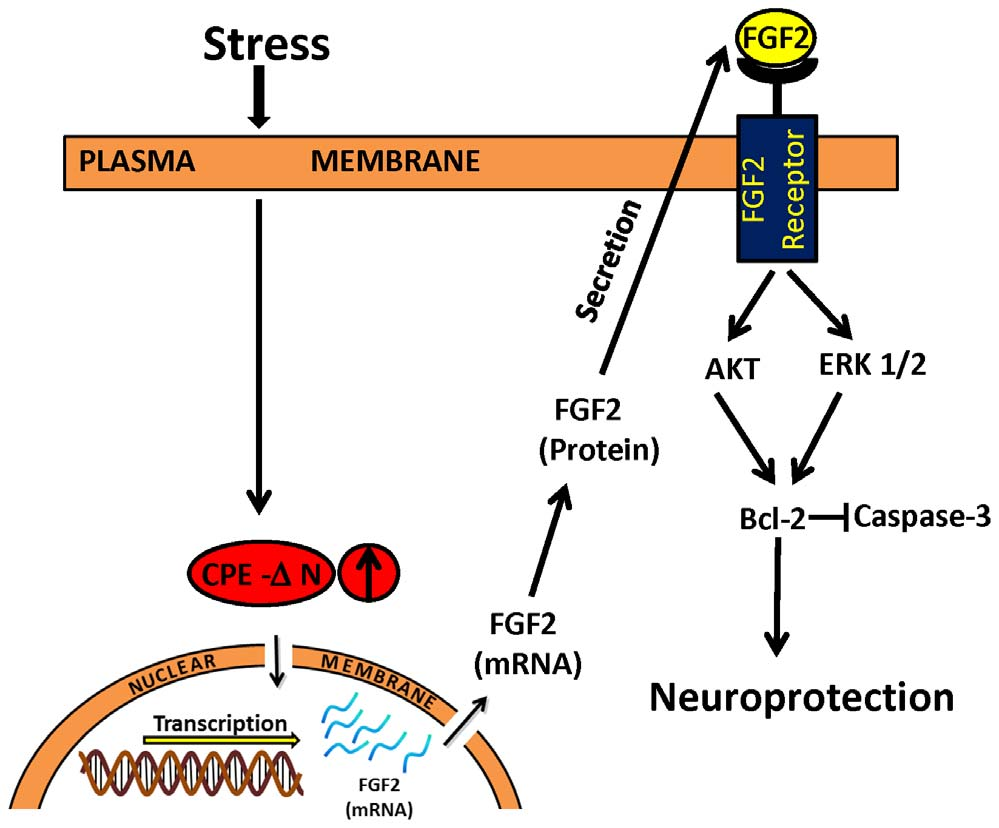
Mechanism of neuroprotection by CPE-ΔN in embryonic neurons during stress. Stress causes release of glucocorticoid which enhances the expression of CPE-ΔN in embryonic neurons. CPE-ΔN is transported into the nucleus and up-regulates the transcription of FGF2 mRNA and subsequent expression of FGF2 protein. The FGF2 is secreted and binds to FGF2 receptors in neurons to activate AKT and ERK signaling pathways which then mediates the up-regulation of expression of BCL-2, an anti-apoptotic protein. BCL-2 inhibits the caspase cascade leading to a decrease in active caspase-3, the apoptotic executioner, thereby resulting in neuroprotection.

In the present study, we found a function for CPE-ΔN in neuroprotection of embryonic neurons against glutamate-induced excitotoxicity potentially caused by maternal stress. Fig. 7 shows a model that summarizes a proposed mechanism of action. We showed that expression of CPE-ΔN in neurons was up-regulated in response to dexamethasone, the synthetic stress hormone (Fig. 2C). *In vivo*, maternal stress causes release of glucocorticoid [38]. Maternal glucocorticoid has been shown to penetrate the placental barrier, with 10–20% reaching the fetus intact [39], and transported to the fetal brain [40]. Excess glucocorticoid has been shown to increase glutamate release and accumulation, leading to neuronal damage [41]. Thus, CPE-ΔN expression in the embryonic neurons could be up-regulated by glucocorticoids as demonstrated by dexamethasone treatment of rat E18 hippocampal neurons (Fig. 2C), or other factors secreted during maternal stress, and play a role in neuroprotection in the fetal brain.

Here we demonstrated that CPE-ΔN can be found in the nucleus of cortical neurons and enhances the transcription of *FGF2* mRNA. FGF2 translated from this mRNA is secreted to serve as a signaling molecule to mediate neuroprotection. Our studies showed that an inhibitor of FGFR1, PD1666285, blocked the neuroprotective effect of CPE-ΔN in glutamate-induced cell death assays, supporting a role of secreted FGF2 in mediating the CPE-ΔN-induced neuroprotection. We showed that overexpression of CPE-ΔN in cortical neurons was 15% of the cell population, yet the neuroprotection of CPE-ΔN against glutamate induced cell death was greater than 15% as shown in Fig. 3. This suggests that widespread protection by CPE-ΔN, likely due to the increased FGF secretion from the transfected cells acting on non-transfected cells to protect them. Studies have shown that FGF2 activates the phosphorylation of AKT and ERK, but only AKT played a role in protecting hippocampal neurons from cell death induced by low insulin in serum-free medium [32]. However, our findings indicate that activation of phosphorylation of both AKT and ERK are involved in CPE-ΔN-mediated neuroprotection against glutamate-induced cytotoxicity. Glutamate-induced neuronal cell death is due to production of reactive oxygen species [42] that causes apoptosis characterized by leakage of cytochrome c from the mitochondria and activation of a cascade of capases including caspase-3, to cause apoptosis [43]. Our study showed that BCL-2, a mitochondrial anti-apoptotic protein and a known down-stream target gene of AKT signaling pathway [44] was up-regulated in expression in the presence of glutamate in cortical neurons overexpressing CPE-ΔN (Fig. 6A). Moreover, activation of caspase-3 was inhibited in these cells treated with glutamate (Fig. 6B). Our findings taken together indicate that CPE-ΔN protects embryonic neurons against glutamate-induced apoptotic cell death through enhancement of FGF2 expression, which then activates the AKT and ERK pathways to up-regulate expression of BCL-2, further leading to inhibition of caspase-3 activation and neuronal survival (Fig. 7).

In summary, our studies have discovered a new neuroprotective protein, CPE-ΔN that is transiently expressed in the mouse embryonic and PN1 brain, but not in adults. CPE-ΔN was found to protect embryonic cortical and hippocampal neurons against cell death induced by glutamate, H_2_O_2_ or staurosporine. CPE-ΔN mediates its neuroprotective effect by enhancing FGF2 expression. FGF2 then acts extracellularly to activate the AKT and ERK signaling pathway which in turn increases BCL-2 expression, leading to inhibition of caspase-3 activity, thereby promoting cell survival. Our findings have implications that CPE-ΔN may play an important role in protecting the fetal brain against neuronal damage induced by reactive oxygen species. CPE-ΔN may have other functions as well during embryonic neurodevelopment besides neuroprotection, such as proliferation and differentiation of neuroprogenitors, since it can activate expression of other genes such as Nedd9 which is involved in cell proliferation and migration in cancer cells [21].

## Supporting Information

Figure S1 **CPE-ΔN protects hippocampal neurons against glutamate-induced cell death.** Rat embryonic primary hippocampal neurons were transduced with adenovirus carrying the CPE-ΔN or LacZ control construct for 72 h and then treated with and without 40 µM glutamate for 24 h. Bar graphs show WST activity indicative of viability of cortical neurons, with and without transduction of CPE-ΔN and with or without treatment with glutamate. Note that the reduced cell viability after glutamate treatment was significantly increased in neurons transduced with the CPE-ΔN construct. Values are mean ± SEM, one-way ANOVA [F_(3,16)_ = 23.54, p<0.001] followed by Tukey test,*p<0.05. Two independent experiments were done. Data shown represent one experiment.
(TIFF)

Figure S2 **CPE-ΔN protects cortical neurons against H_2_O_2_-induced cell death.** In A–D, rat embryonic cortical neurons were transduced with adenovirus carrying the CPE-ΔN or LacZ control construct for 72 h and then treated with and without 100 µM H_2_O_2_ for 24 h. **A.** Bar graphs show WST activity indicative of viability of cortical neurons, with or without treatment with H_2_O_2_. Note that the reduced cell viability after H_2_O_2_ treatment was significantly increased in neurons transduced with the CPE-ΔN construct. At least three independent experiments were done. Data shown represent one experiment. **B.** Bar graphs show LDH release, indicative of cytotoxicity of cortical neurons treated with or without H_2_O_2_. Note that the H_2_O_2_-induced cell death was significantly attenuated by the transduction of CPE-ΔN construct. At least three independent experiments were done. Data shown represent one experiment. **C, D.** Photomicrographs and bar graphs showing cortical neurons with or without H_2_O_2_ treatment and stained with TUNEL (green) and DAPI (blue). Note that the number of dead cells (green) increased significantly after H_2_O_2_ treatment and that transduction of CPE-ΔN construct protected the neurons. The bar graphs represent the quantification of dead cells as a % of the total number of cells determined by the DAPI staining. At least 500 cells were counted in each of 3 different dish generated from embryos from two independent experiments. Data shown represent one experiment. Bar = 100 microns. (A, B, D) Values are mean ± SEM, one-way ANOVA followed by Tukey test, *p<0.05. A: ANOVA, F_(3,36)_ = 31.54, P<0.001; B: ANOVA, F_(3,36)_ = 185.6, P<0.001. D: ANOVA, F_(3,8)_ = 245.5. P<0.001.
(TIFF)

Figure S3 **CPE-ΔN protects cortical neurons against staurosporine-induced cell death.** In A–D, rat embryonic cortical neurons were transduced with adenovirus carrying the CPE-ΔN or LacZ control construct for 72 h and then treated with and without 0.4 µM staurosporine (STS) for 24 h. **A.** Bar graphs show WST activity, indicative of cell viability of cortical neurons with or without treatment with staurosporine. Note that the reduced cell viability after staurosporine treatment was significantly increased in neurons transduced with the CPE-ΔN construct. At least three independent experiments were done. Data shown represent one experiment. **B.** Bar graphs show LDH release, indicative of cytotoxicity of cortical neurons treated with or without H_2_O_2_. Note that the staurosporine -induced cell death was significantly attenuated by the transduction of CPE-ΔN construct. At least three independent experiments were done. Data shown represent one experiment. **C, D.** Photomicrographs and bar graphs showing cortical neurons with or without staurosporine treatment and stained with TUNEL (green) and DAPI (blue). Note that the number of dead cells (green) increased significantly after glutamate treatment and that transduction of CPE-ΔN construct protected the neurons. The bar graphs represent the quantification of dead cells as a % of the total number of cells determined by the DAPI staining. At least 500 cells were counted in each of 3 different dish generated from embryos from two independent experiments. Data shown represent one experiment. Bar = 100 microns. (A, B, D): Values are mean ± SEM, one-way ANOVA followed by Tukey test, *p<0.05. A: ANOVA, F_(3,20)_ = 19.05, P<0.001; B: ANOVA, F_(3,16)_ = 29.57, P<0.001; D: ANOVA, F_(3,8)_ = 115.7, P<0.001.
(TIFF)

Figure S4 **Neuroprotection by CPE-ΔN against H_2_O_2_-induced cell death is mediated by FGF2.** In A–C, rat primary cortical neurons were transduced with CPE-ΔN or LacZ viral construct for 72 h and subsequently treated with or without 100 µM H_2_O_2_ for 24 h. **A.** Top panel: Western blot analysis of FGF2 protein in primary cortical neurons treated with or without H_2_O_2_. Actin was also analyzed and served as an internal control for protein load; Bottom panel: Bar graphs show the quantification of FGF2 protein normalized to actin and expressed as a % compared to vehicle treated control cells. Note that CPE-ΔN significantly inhibited the H_2_O_2_-induced decrease in FGF2 protein in the cortical neurons. At least three independent experiments were done. Data shown represent all the experiments combined. **B.** Bar graphs show WST activity, indicative of cell viability of cortical neurons treated with and without H_2_O_2_ in the continued presence or absence of FGF receptor inhibitor, PD166285. Two independent experiments were done. Data shown represent one experiment. **C.** Bar graphs show WST activity, indicative of cell viability of cortical neurons treated with and without H_2_O_2_ in the continued presence or absence of FGF receptor inhibitor, SU5402. One experiment was done. Note the neuroprotective effect of CPE-ΔN was blocked by PD166285 and SU5402 in primary cortical neurons, indicating that FGF2 mediates the effect. (A, B, C) Values are mean ± SEM, one-way ANOVA followed by Tukey test,*p<0.05. A: ANOVA: F_(3,20)_ = 11.7, p<0.001; B: ANOVA: F_(7,32)_ = 24.7, P<0.001. C: ANOVA: F_(7,32)_ = 10.08, p<0.001.
(TIFF)

Figure S5 **Neuroprotection by CPE-ΔN against staurosporine-induced cell death is mediated by FGF2.** In A–D, rat primary cortical neurons were transduced with CPE-ΔN or LacZ viral construct and subsequently treated with or without 0.4 µM staurosporine for 24 h. **A.** Top panel: Western blot analysis of FGF2 protein in primary cortical neurons, treated with or without staurosporine (STS). Actin was also analyzed and served as an internal control for protein load; Bottom panel: Bar graphs show the quantification of FGF2 protein normalized to actin and expressed as a % compared to vehicle treated control cells. Note that CPE-ΔN significantly inhibited the staurosporine-induced decrease in FGF2 protein in the cortical neurons. At least three independent experiments were done. Data shown represent all the experiments combined. **B, C.** Bar graphs show WST activity, indicative of cell viability of cortical neurons treated with and without staurosporine in the continued presence or absence of FGF receptor inhibitors, PD166285 (B, two independent experiments were done, data shown represent one experiment), or SU5402 (C, one experiment was done). Note the neuroprotective effect of CPE-ΔN was blocked by PD166285 and SU5402 in the cortical neurons, indicating that FGF2 mediates the effect. (A–C) Values are mean ± SEM, one-way ANOVA followed by Tukey test,*p<0.05. A: ANOVA, F_(3,20)_ = 15.56, p<0.001; B: ANOVA: F_(7,32)_ = 80.38, P<0.001. C: ANOVA: F_(7,32)_ = 5.618, P<0.001.
(TIFF)

Figure S6 **Specificity of FGF2 receptor inhibitor in cortical neurons.** In A–B, rat primary cortical neurons were treated with vehicle or PD166285 and subsequently treated with FGF2 or BDNF. **A.** Top panel: Western blot analysis of p-ERK in cortical neurons after various treatments. t-ERK was also analyzed and served as an internal control for protein load; bottom panel: Bar graphs showing the quantification of p-ERK normalized to t-ERK and expressed as a % compared to vehicle treated control cells. **B.** Top panel: Western blot analysis of p-AKT in cortical neurons after various treatments. t-AKT was also analyzed and served as an internal control for protein load; bottom panel: Bar graphs showing the quantification of p-AKT normalized to t-AKT and expressed as a % compared to vehicle treated control cells. Note PD166285 blocked the activation of ERK or AKT by exogenous FGF2 but not by BDNF. Data was from one experiment with three samples for each group. (A–B) Values are mean ± SEM, one-way ANOVA followed by Tukey test,*p<0.05. A: ANOVA, F_(4,14)_ = 18.01, p<0.001; A: ANOVA, F_(4,14)_ = 15.53, p<0.001.
(TIFF)

Figure S7 **Neuroprotection by CPE-ΔN against H_2_O_2_-induced cell death involves.** **AKT and ERK signaling pathways.** In A–D, rat primary cortical neurons were transduced with CPE-ΔN or LacZ viral construct and subsequently treated with or without 100 µM H_2_O_2_ for 24 h. **A.** Top panel: Western blot analysis of p-AKT protein in primary cultured cortical neurons treated with or without H_2_O_2_. Actin was also analyzed and served as an internal control for protein load. Bottom panel: Bar graphs showing the quantification of p-AKT normalized to actin and expressed as a % compared to vehicle treated control cells. Note that CPE-ΔN significantly inhibited the H_2_O_2_-induced decrease in p-AKT in the cortical neurons. At least three independent experiments were done. Data shown represent all the experiments combined. **B.** Bar graphs showing WST activity, indicative of cell viability, of cortical neurons treated with and without H_2_O_2_ in the continued presence or absence of AKT inhibitor, Ly294002. Note the neuroprotective effect of CPE-ΔN was completely blocked by Ly294002 in the cortical neurons, suggesting the AKT signaling pathway is involved. Two independent experiments were done. Data shown represent one experiment. **C.** Top panel: Western blot analysis of p-ERK in cortical neurons treated with or without H_2_O_2_. Actin was also analyzed and served as an internal control for protein load; bottom panel: Bar graphs showing the quantification of p-ERK normalized to actin and expressed as a % compared to vehicle treated control cells. Note that CPE-ΔN significantly inhibited the H_2_O_2_-induced decrease in p-ERK in the cortical neurons. At least three independent experiments were done. Data shown represent all the experiments combined. **D.** Bar graphs showing WST activity, indicative of cell viability of cortical neurons treated with and without H_2_O_2_ in the continued presence or absence of the ERK inhibitor, U0126. Note the neuroprotective effect of CPE-ΔN was partially blocked by U0126, suggesting the involvement of ERK signaling pathway. Two independent experiments were done. Data shown represent one experiment. (A–D) Values are mean ± SEM, one-way ANOVA followed by Tukey test,*p<0.05. A: ANOVA: F_(3,12)_ = 47.1, P<0.001; B: ANOVA: F_(5,24)_ = 41.7, P<0.001. C: ANOVA: F_(3,20)_ = 8.581, P<0.001; D: ANOVA: F_(7,32)_ = 8.367, P<0.001.
(TIFF)
