# Supplementary figures and images for: Carboxypeptidase E-ΔN, a Neuroprotein Transiently Expressed during Development Protects Embryonic Neurons against Glutamate Neurotoxicity
Source: PLoS One. 2014 Nov 26;9(11):e112996. doi: 10.1371/journal.pone.0112996 (PMC4245097; doi:10.1371/journal.pone.0112996)

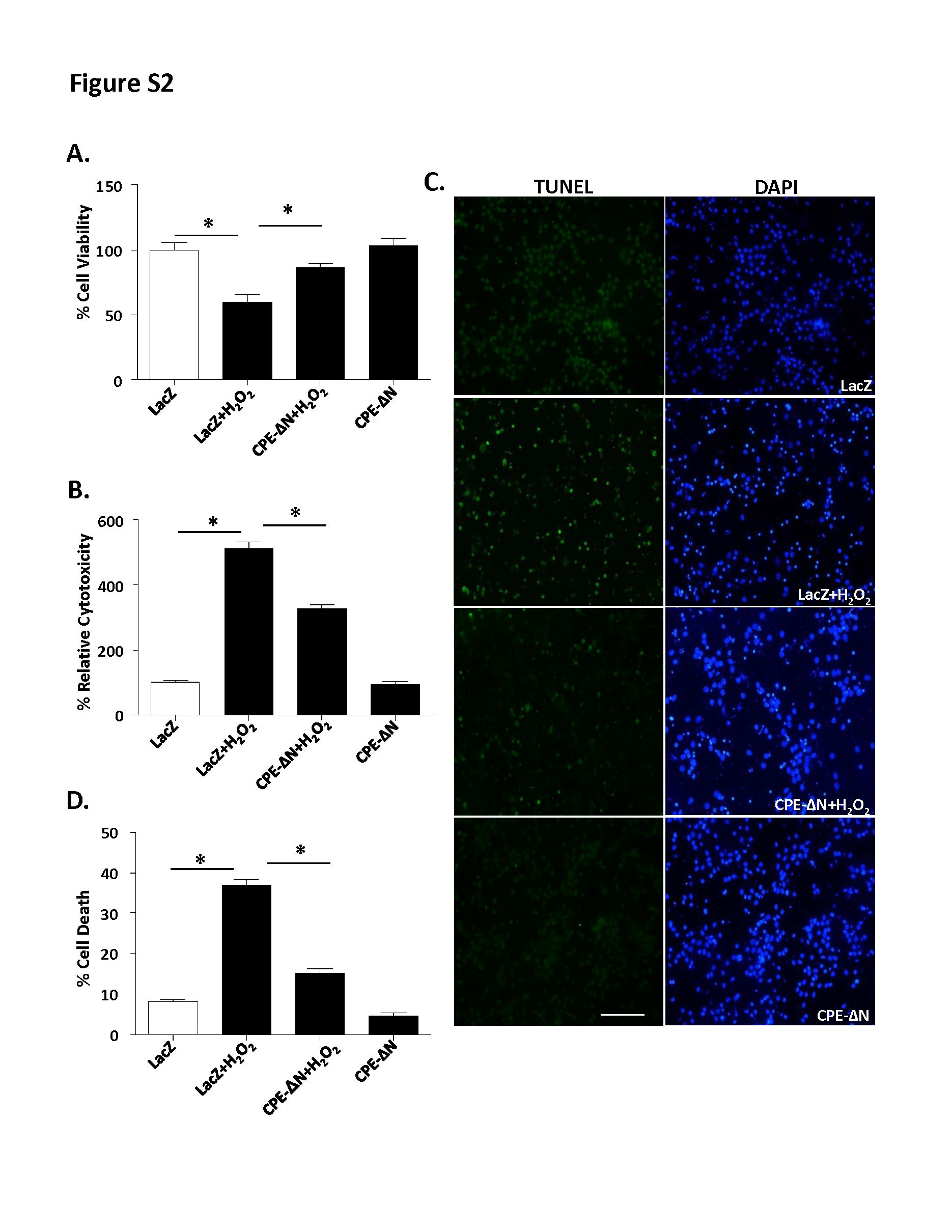

Supplement: Figure S2 — CPE-ΔN protects cortical neurons against H2O2-induced cell death. In A–D, rat embryonic cortical neurons were transduced with adenovirus carrying the CPE-ΔN or LacZ control construct for 72 h and then treated with and without 100 µM H2O2 for 24 h. A. Bar graphs show WST activity indicative of viability of cortical neurons, with or without treatment with H2O2. Note that the reduced cell viability after H2O2 treatment was significantly increased in neurons transduced with the CPE-ΔN construct. At least three independent experiments were done. Data shown represent one experiment. B. Bar graphs show LDH release, indicative of cytotoxicity of cortical neurons treated with or without H2O2. Note that the H2O2-induced cell death was significantly attenuated by the transduction of CPE-ΔN construct. At least three independent experiments were done. Data shown represent one experiment. C, D. Photomicrographs and bar graphs showing cortical neurons with or without H2O2 treatment and stained with TUNEL (green) and DAPI (blue). Note that the number of dead cells (green) increased significantly after H2O2 treatment and that transduction of CPE-ΔN construct protected the neurons. The bar graphs represent the quantification of dead cells as a % of the total number of cells determined by the DAPI staining. At least 500 cells were counted in each of 3 different dish generated from embryos from two independent experiments. Data shown represent one experiment. Bar = 100 microns. (A, B, D) Values are mean ± SEM, one-way ANOVA followed by Tukey test, *p<0.05. A: ANOVA, F(3,36) = 31.54, P<0.001; B: ANOVA, F(3,36) = 185.6, P<0.001. D: ANOVA, F(3,8) = 245.5. P<0.001. (TIFF) [file pone.0112996.s002.tiff]

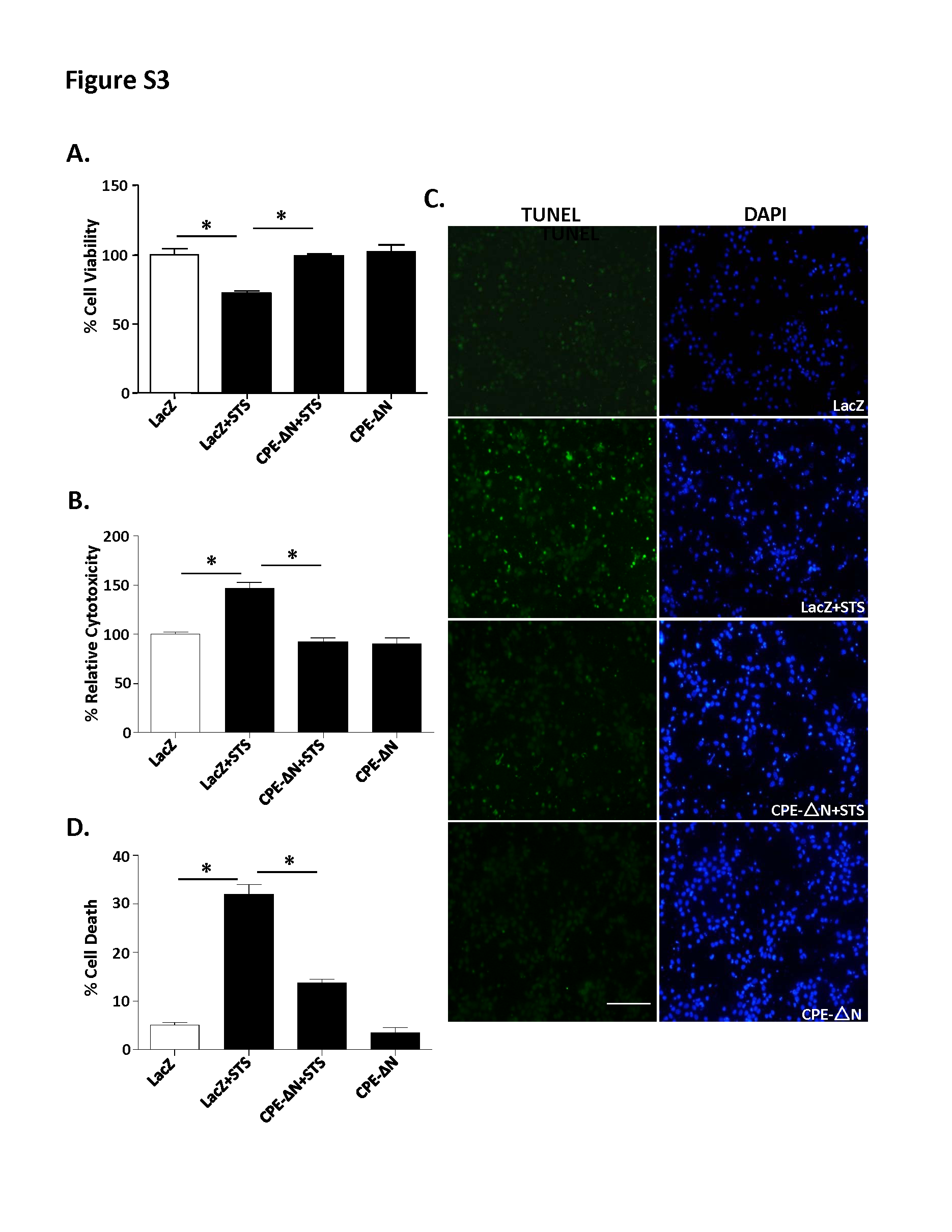

Supplement: Figure S3 — CPE-ΔN protects cortical neurons against staurosporine-induced cell death. In A–D, rat embryonic cortical neurons were transduced with adenovirus carrying the CPE-ΔN or LacZ control construct for 72 h and then treated with and without 0.4 µM staurosporine (STS) for 24 h. A. Bar graphs show WST activity, indicative of cell viability of cortical neurons with or without treatment with staurosporine. Note that the reduced cell viability after staurosporine treatment was significantly increased in neurons transduced with the CPE-ΔN construct. At least three independent experiments were done. Data shown represent one experiment. B. Bar graphs show LDH release, indicative of cytotoxicity of cortical neurons treated with or without H2O2. Note that the staurosporine -induced cell death was significantly attenuated by the transduction of CPE-ΔN construct. At least three independent experiments were done. Data shown represent one experiment. C, D. Photomicrographs and bar graphs showing cortical neurons with or without staurosporine treatment and stained with TUNEL (green) and DAPI (blue). Note that the number of dead cells (green) increased significantly after glutamate treatment and that transduction of CPE-ΔN construct protected the neurons. The bar graphs represent the quantification of dead cells as a % of the total number of cells determined by the DAPI staining. At least 500 cells were counted in each of 3 different dish generated from embryos from two independent experiments. Data shown represent one experiment. Bar = 100 microns. (A, B, D): Values are mean ± SEM, one-way ANOVA followed by Tukey test, *p<0.05. A: ANOVA, F(3,20) = 19.05, P<0.001; B: ANOVA, F(3,16) = 29.57, P<0.001; D: ANOVA, F(3,8) = 115.7, P<0.001. (TIFF) [file pone.0112996.s003.tiff]

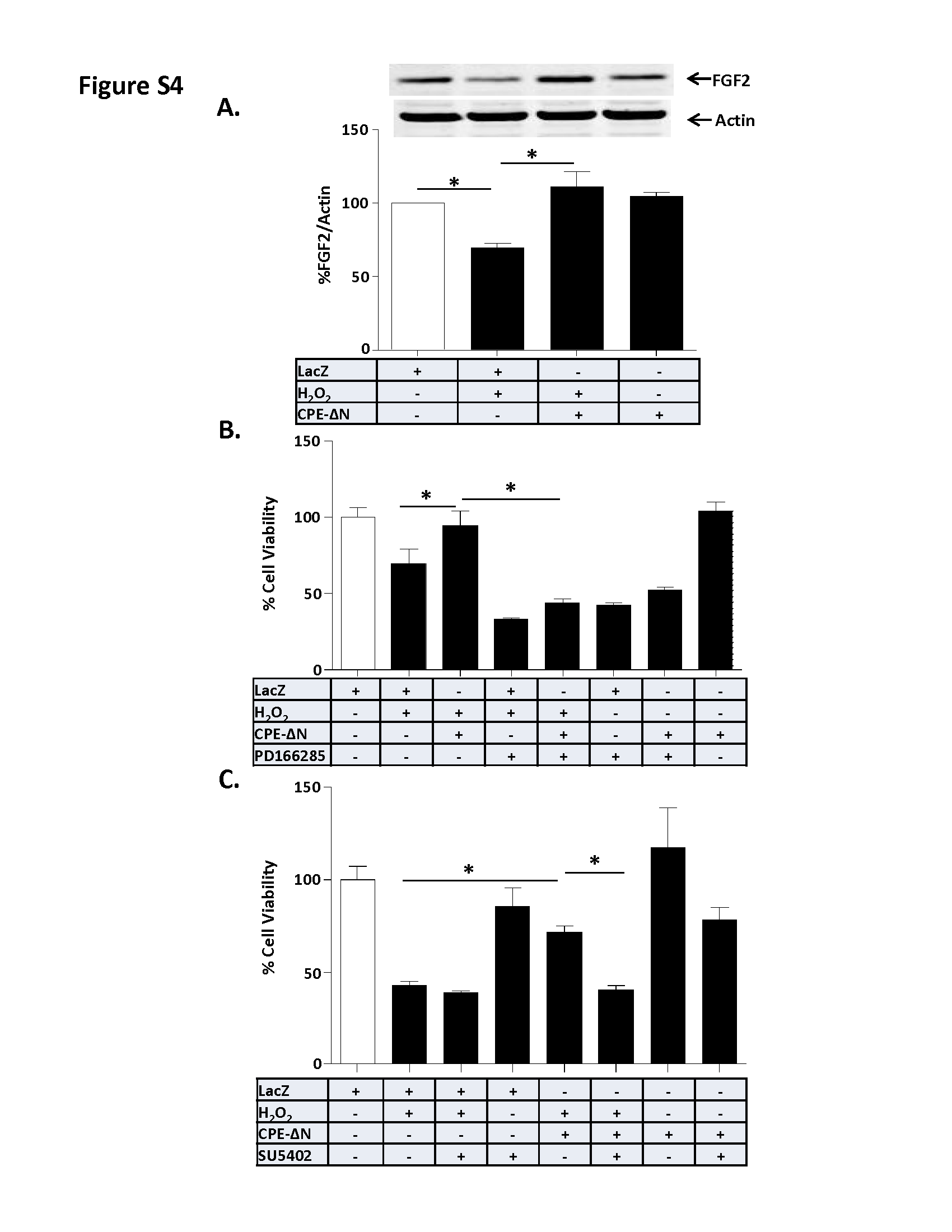

Supplement: Figure S4 — Neuroprotection by CPE-ΔN against H2O2-induced cell death is mediated by FGF2. In A–C, rat primary cortical neurons were transduced with CPE-ΔN or LacZ viral construct for 72 h and subsequently treated with or without 100 µM H2O2 for 24 h. A. Top panel: Western blot analysis of FGF2 protein in primary cortical neurons treated with or without H2O2. Actin was also analyzed and served as an internal control for protein load; Bottom panel: Bar graphs show the quantification of FGF2 protein normalized to actin and expressed as a % compared to vehicle treated control cells. Note that CPE-ΔN significantly inhibited the H2O2-induced decrease in FGF2 protein in the cortical neurons. At least three independent experiments were done. Data shown represent all the experiments combined. B. Bar graphs show WST activity, indicative of cell viability of cortical neurons treated with and without H2O2 in the continued presence or absence of FGF receptor inhibitor, PD166285. Two independent experiments were done. Data shown represent one experiment. C. Bar graphs show WST activity, indicative of cell viability of cortical neurons treated with and without H2O2 in the continued presence or absence of FGF receptor inhibitor, SU5402. One experiment was done. Note the neuroprotective effect of CPE-ΔN was blocked by PD166285 and SU5402 in primary cortical neurons, indicating that FGF2 mediates the effect. (A, B, C) Values are mean ± SEM, one-way ANOVA followed by Tukey test,*p<0.05. A: ANOVA: F(3,20) = 11.7, p<0.001; B: ANOVA: F(7,32) = 24.7, P<0.001. C: ANOVA: F(7,32) = 10.08, p<0.001. (TIFF) [file pone.0112996.s004.tiff]

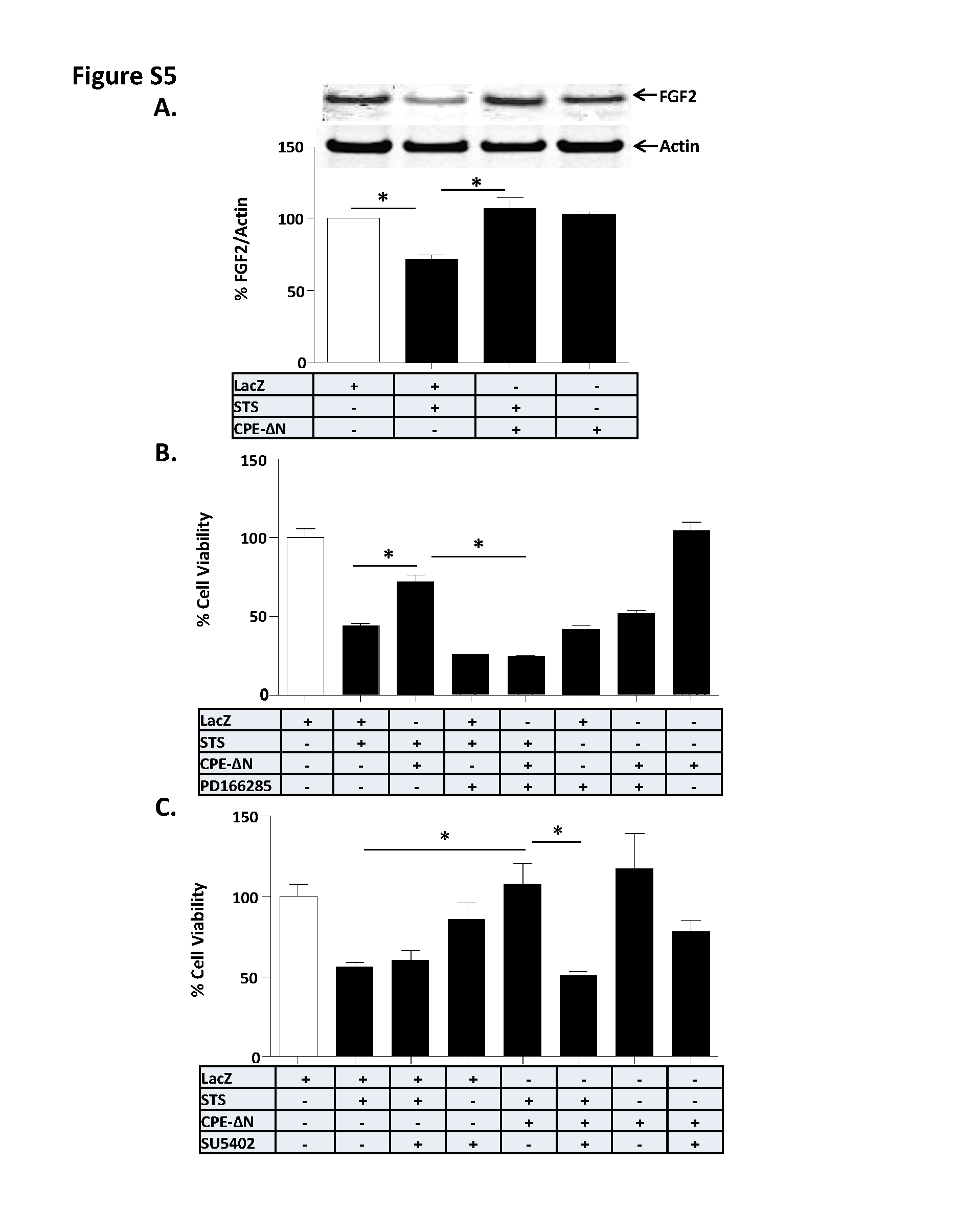

Supplement: Figure S5 — Neuroprotection by CPE-ΔN against staurosporine-induced cell death is mediated by FGF2. In A–D, rat primary cortical neurons were transduced with CPE-ΔN or LacZ viral construct and subsequently treated with or without 0.4 µM staurosporine for 24 h. A. Top panel: Western blot analysis of FGF2 protein in primary cortical neurons, treated with or without staurosporine (STS). Actin was also analyzed and served as an internal control for protein load; Bottom panel: Bar graphs show the quantification of FGF2 protein normalized to actin and expressed as a % compared to vehicle treated control cells. Note that CPE-ΔN significantly inhibited the staurosporine-induced decrease in FGF2 protein in the cortical neurons. At least three independent experiments were done. Data shown represent all the experiments combined. B, C. Bar graphs show WST activity, indicative of cell viability of cortical neurons treated with and without staurosporine in the continued presence or absence of FGF receptor inhibitors, PD166285 (B, two independent experiments were done, data shown represent one experiment), or SU5402 (C, one experiment was done). Note the neuroprotective effect of CPE-ΔN was blocked by PD166285 and SU5402 in the cortical neurons, indicating that FGF2 mediates the effect. (A–C) Values are mean ± SEM, one-way ANOVA followed by Tukey test,*p<0.05. A: ANOVA, F(3,20) = 15.56, p<0.001; B: ANOVA: F(7,32) = 80.38, P<0.001. C: ANOVA: F(7,32) = 5.618, P<0.001. (TIFF) [file pone.0112996.s005.tiff]

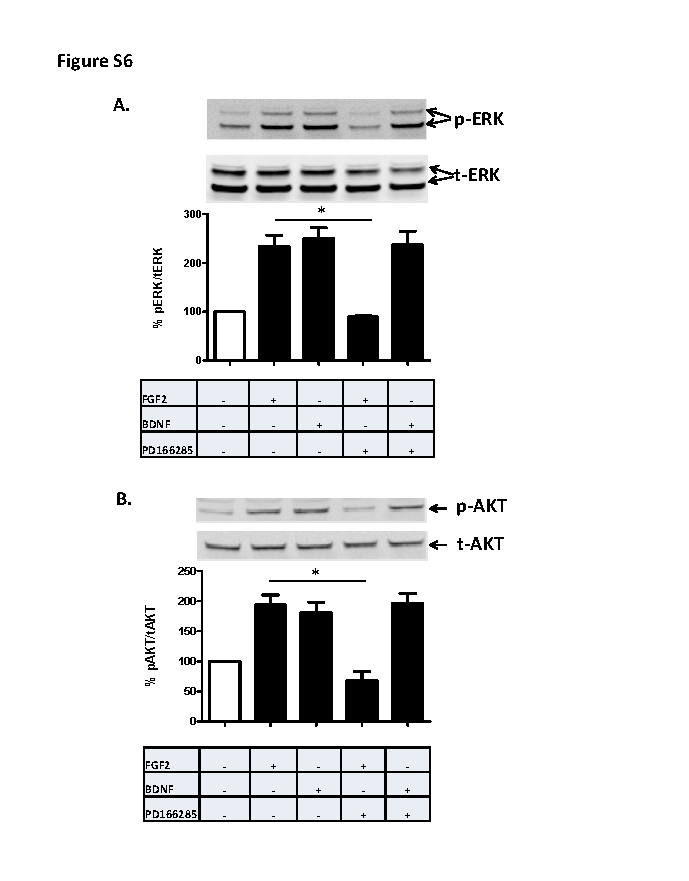

Supplement: Figure S6 — Specificity of FGF2 receptor inhibitor in cortical neurons. In A–B, rat primary cortical neurons were treated with vehicle or PD166285 and subsequently treated with FGF2 or BDNF. A. Top panel: Western blot analysis of p-ERK in cortical neurons after various treatments. t-ERK was also analyzed and served as an internal control for protein load; bottom panel: Bar graphs showing the quantification of p-ERK normalized to t-ERK and expressed as a % compared to vehicle treated control cells. B. Top panel: Western blot analysis of p-AKT in cortical neurons after various treatments. t-AKT was also analyzed and served as an internal control for protein load; bottom panel: Bar graphs showing the quantification of p-AKT normalized to t-AKT and expressed as a % compared to vehicle treated control cells. Note PD166285 blocked the activation of ERK or AKT by exogenous FGF2 but not by BDNF. Data was from one experiment with three samples for each group. (A–B) Values are mean ± SEM, one-way ANOVA followed by Tukey test,*p<0.05. A: ANOVA, F(4,14) = 18.01, p<0.001; A: ANOVA, F(4,14) = 15.53, p<0.001. (TIFF) [file pone.0112996.s006.tiff]

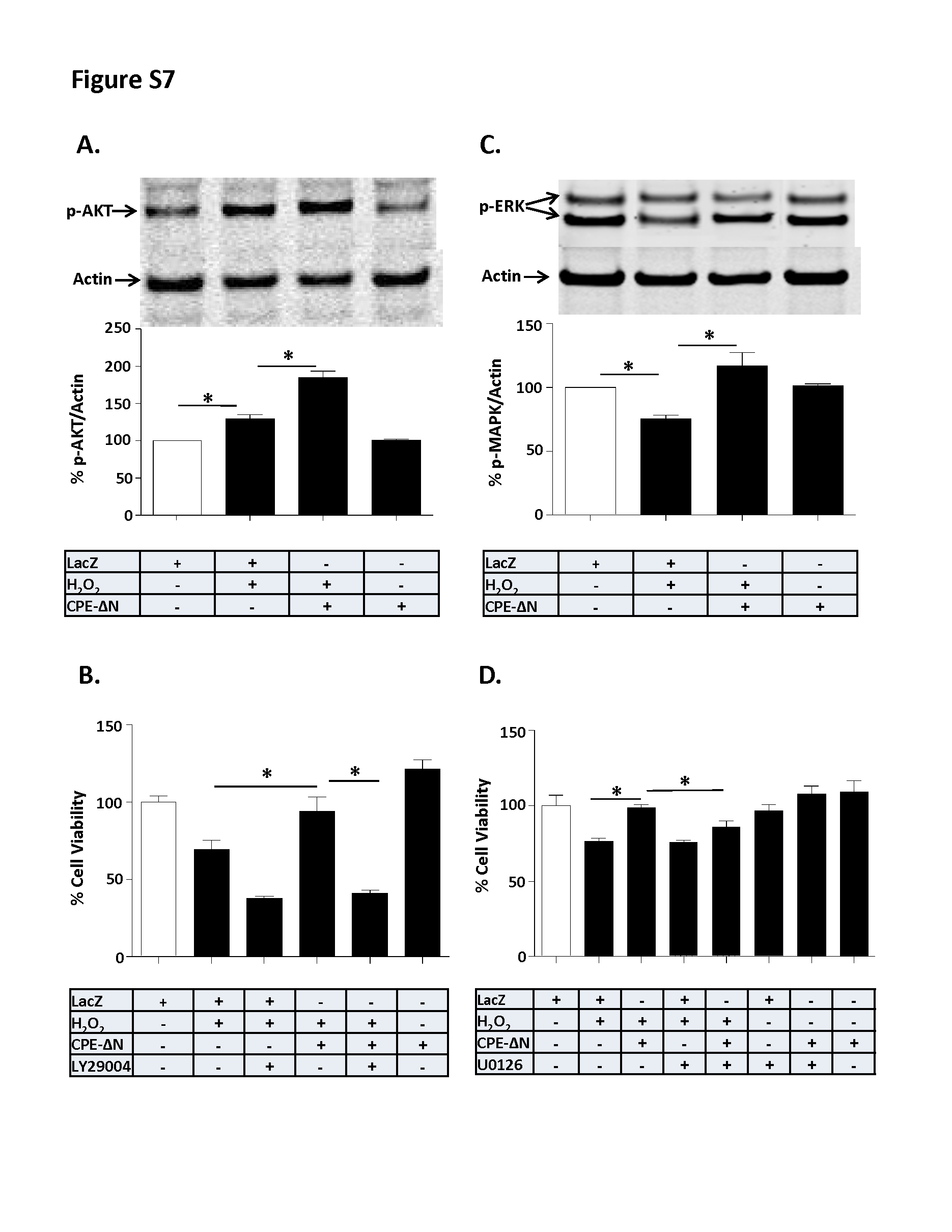

Supplement: Figure S7 — Neuroprotection by CPE-ΔN against H2O2-induced cell death involves. AKT and ERK signaling pathways. In A–D, rat primary cortical neurons were transduced with CPE-ΔN or LacZ viral construct and subsequently treated with or without 100 µM H2O2 for 24 h. A. Top panel: Western blot analysis of p-AKT protein in primary cultured cortical neurons treated with or without H2O2. Actin was also analyzed and served as an internal control for protein load. Bottom panel: Bar graphs showing the quantification of p-AKT normalized to actin and expressed as a % compared to vehicle treated control cells. Note that CPE-ΔN significantly inhibited the H2O2-induced decrease in p-AKT in the cortical neurons. At least three independent experiments were done. Data shown represent all the experiments combined. B. Bar graphs showing WST activity, indicative of cell viability, of cortical neurons treated with and without H2O2 in the continued presence or absence of AKT inhibitor, Ly294002. Note the neuroprotective effect of CPE-ΔN was completely blocked by Ly294002 in the cortical neurons, suggesting the AKT signaling pathway is involved. Two independent experiments were done. Data shown represent one experiment. C. Top panel: Western blot analysis of p-ERK in cortical neurons treated with or without H2O2. Actin was also analyzed and served as an internal control for protein load; bottom panel: Bar graphs showing the quantification of p-ERK normalized to actin and expressed as a % compared to vehicle treated control cells. Note that CPE-ΔN significantly inhibited the H2O2-induced decrease in p-ERK in the cortical neurons. At least three independent experiments were done. Data shown represent all the experiments combined. D. Bar graphs showing WST activity, indicative of cell viability of cortical neurons treated with and without H2O2 in the continued presence or absence of the ERK inhibitor, U0126. Note the neuroprotective effect of CPE-ΔN was partially blocked by U0126, suggesting the involvem [file pone.0112996.s007.tiff]
